# Drivers of human *Leptospira* infection in the Pacific Islands: A systematic review

**DOI:** 10.1017/S0950268824001250

**Published:** 2024-10-08

**Authors:** Sahil Kharwadkar, Philip Weinstein, Jessica Stanhope

**Affiliations:** 1 School of Public Health, The University of Adelaide, Adelaide, Australia; 2 Adelaide Medical School, The University of Adelaide, Adelaide, Australia; 3 School of Allied Health Science and Practice, The University of Adelaide, Adelaide, Australia

**Keywords:** leptospirosis, public health, tropical diseases, zoonoses, Pacific Islands

## Abstract

Leptospirosis is a bacterial zoonosis that poses an increasing global public health risk. Pacific Island communities are highly vulnerable to leptospirosis outbreaks, yet the local drivers of infection remain poorly understood. We conducted a systematic review to identify the drivers of human *Leptospira* infection in the Pacific Islands. There were 42 included studies from which findings were synthesized descriptively. In tropical Pacific Islands, infections were a product of sociodemographic factors such as male gender/sex, age 20 to 60 years, Indigenous ethnicity, and poverty; lifestyle factors such as swimming, gardening, and open skin wounds; and environmental factors, including seasonality, heavy rainfall, and exposure to rodents, cattle, and pigs. Possible mitigation strategies in these islands include strengthening disease reporting standards at a regional level; improving water security, rodent control, and piggery management at a community level; and information campaigns to target individual-level drivers of infection. By contrast, in New Zealand, exposures were predominantly occupational, with infections occurring in meat and farm workers. Accordingly, interventions could include adjustments to occupational practices and promoting the uptake of animal vaccinations. Given the complexity of disease transmission and future challenges posed by climate change, further action is required for leptospirosis control in the Pacific Islands.

## Introduction

Human leptospirosis is a bacterial zoonotic infection that accounts for approximately one million cases and 60,000 deaths globally each year [1]. Leptospirosis is caused by spirochete bacteria, *Leptospira*, which are most often carried by rodents, livestock, and dogs [2]. Leptospires are excreted via animal urine into the environment, where humans may become infected through contact with infected animals or contaminated soil and water [2]. An increasing number of leptospirosis outbreaks have been reported around the world, particularly following heavy rainfall and flooding [3].

Human leptospirosis poses a significant public health risk in the Pacific Islands, with Oceania representing the highest burden of leptospirosis worldwide, as measured by morbidity (150.68 cases per 100,000 per year) and mortality (9.61 deaths per 100,000 per year) [1]. Climate change presents an additional threat to leptospirosis burden, with flooding predicted to become more intense and frequent in the region [4]. However, there is currently limited knowledge about the primary drivers of human *Leptospira* infection in the Pacific Islands. The local incidence of leptospirosis remains poorly documented due to the unavailability of laboratory diagnosis, limited medical awareness, and non-specific symptoms that overlap with other tropical diseases [5]. Consequently, it has been difficult for local authorities to implement mitigation strategies [5].

Whilst an existing review examined the drivers of leptospirosis at a broader scale [6], many Pacific Island studies were excluded from their analysis. As a result, their synthesized findings are not necessarily applicable to the context of these vulnerable islands. By better understanding the local drivers of infection within the region, appropriate mitigation measures can be implemented to combat leptospirosis outbreaks.

Hence, the aim of this systematic review was to identify the drivers of human *Leptospira* infection in the Pacific Islands, which will lead to recommendations for public health interventions to reduce future disease burden. We have considered the Pacific Islands to refer to the three ethnogeographic groups of Melanesia, Micronesia, and Polynesia, which includes New Zealand but excludes neighbouring islands of Australia, Indonesia, and the Philippines [7].

## Materials and methods

This systematic review has been reported according to the Preferred Reporting Items for Systematic Reviews and Meta-Analyses (PRISMA) statement [8].

### Search strategy

Ovid Medline (1946–present), Scopus (1960–present), Web of Science Core Collection (1900–present), and Ovid Embase (1974–present) electronic databases were searched in December 2022 using search terms in the title, author keyword, and abstract fields. The search also included subject headings in Embase and Medline (i.e. Emtree and Medical Subject Headings (MeSH), respectively), as well as indexed terms for Scopus (i.e. indexed keywords) and Web of Science Core Collection (i.e. Keywords Plus) (see Supplementary Material S1 for details). There were no language or date restrictions. The reference and citation lists (Web of Science Core Collection, Scopus, and Google Scholar) of included studies, as well as relevant excluded reviews, were also screened for additional studies. Reference and citation screening were performed independently by two reviewers (S.K. and J.S.) in June 2023.

### Study selection

All identified articles were exported into Covidence, where duplicates were manually and independently removed by two reviewers (S.K. and J.S. for English language studies and S.K. and P.W. for French language studies). The reviewers then independently screened the articles by title and abstract, and full text, according to the eligibility criteria (see Table 1).

**Table 1.** Eligibility criteria for study inclusion using the population, exposure, comparator, and outcomes (PECO) framework [9]

Domain	Criteria
Population	Humans in the Pacific Islands,^a^ including both residents and visitors with no restrictions by age or sex/gender
Exposure	Any potential driver of human *Leptospira* infection investigated using inferential statistics, including but not limited to sociodemographic factors (e.g. gender/sex), occupational (e.g. job), lifestyle (e.g. freshwater contact), and environmental factors (e.g. rainfall)
Comparator	Any relevant comparison (e.g. different or no exposure to the respective driver)
Outcomes	Measures of human *Leptospira* infection, such as incidence and prevalence (including seroprevalence) with laboratory diagnosis, including but not limited to culture, microscopic agglutination test (MAT), polymerase chain reaction (PCR), and enzyme-linked immunosorbent assay (ELISA)^b^
Other criteria	Epidemiological studies, including cross-sectional, case–control, cohort, time series, ecological studies, and case series, which were published in full-text in a peer–reviewed journal^c^

a The following Pacific Islands or regions were included, representing Melanesia, Micronesia, and Polynesia: Fiji, New Caledonia, Papua New Guinea, Solomon Islands, and Vanuatu (Melanesia); Guam, Kiribati, Marshall Islands, Federated States of Micronesia, Nauru, Northern Mariana Islands, Palau, and the US minor outlying islands, which include Baker Island, Howland Island, Jarvis Island, Johnston Atoll, Kingman Reef, Midway Atoll, Palmyra Atoll, and Wake Island (Micronesia); and American Samoa, New Zealand, Cook Islands, Easter Island, Hawaii, Niue, Pitcairn, Samoa, Tokelau, Tonga, Tuvalu, Wallis, Futuna, and French Polynesia, which include the Society Islands, Tuamotu Archipelago, Gambier Islands, Marquesas Islands, and Austral Islands (Polynesia) [10].

b Hospitalization, mortality, and other possible outcomes or complications of *Leptospira* infection were not examined in this review.

c Grey literature, predictive models, reviews, conference abstracts, editorials, and book chapters were excluded.

Any discrepancies that arose were resolved through discussion and consensus decision between the two reviewers, with a third reviewer consulted when consensus was not reached.

### Data extraction

For each included article, two reviewers (S.K. and J.S. for English language studies and S.K. and P.W. for French language studies) manually and independently extracted data into an Excel spreadsheet with the following headings: (a) citation details; (b) study location; (c) year(s) and month(s) of study data collection; (d) study design; (e) population and sample characteristics; (f) driver(s) assessed, how they were measured, and confounders considered; (g) leptospirosis outcome(s) and how they were measured; (h) serogroup(s) and method of identification; (i) statistical approaches; and (j) findings related to the association between the driver(s) and leptospirosis outcome(s).

### Risk-of-bias assessment

Each study was allocated to a level of evidence according to the Australian National Health and Medical Research Council (NHMRC) evidence hierarchy for aetiological studies [11]. The NHMRC evidence hierarchy consists of levels of evidence from I to IV, where level IV represents the study design least robust at answering a research question. As ecological and test-negative case–control study designs are not included in the existing evidence hierarchy, we classified these studies as level IV.

The risk of bias for each study was further evaluated independently by two reviewers (S.K. and J.S. for English language studies and S.K. and P.W. for French language studies) using the validated ‘Methodological Evaluation of Observational Research Checklist’ (MEVORECH) tool for risk factor studies [12] (see Supplementary Table S1 for details). Discrepancies were resolved through discussion and consensus decision between the two reviewers (S.K. and J.S.), with a third reviewer (P.W.) consulted when consensus was not reached.

### Synthesis of results

After data extraction, drivers were classified into the following categories: sociodemographic, occupational, lifestyle, and environmental. Activities performed at a recognized occupational setting, such as an abattoir or farm, were classified as occupational, whereas activities conducted recreationally or at home, including backyard gardening/farming, were considered as lifestyle drivers of infection. Study characteristics and findings were synthesized descriptively, using tables and narrative descriptions. Findings were considered statistically significant if *P* < 0.05 or if the authors reported the finding as significant in studies where no *P*-value was reported. Both unadjusted and adjusted findings are reported for included studies; however, when reporting the number of studies with significant findings, the adjusted estimates have been used. Quantitative synthesis was not performed due to heterogeneity across studies with respect to populations, exposures, comparators, and outcomes.

### Protocol registration

The review protocol was registered prospectively with International Prospective Register of Systematic Reviews (PROSPERO) (CRD42022360109) [13]. Modifications were made to the initial protocol prior to database searches to include the Pacific Islands described in Table 1.

## Results

### Study selection

**Figure 1.**
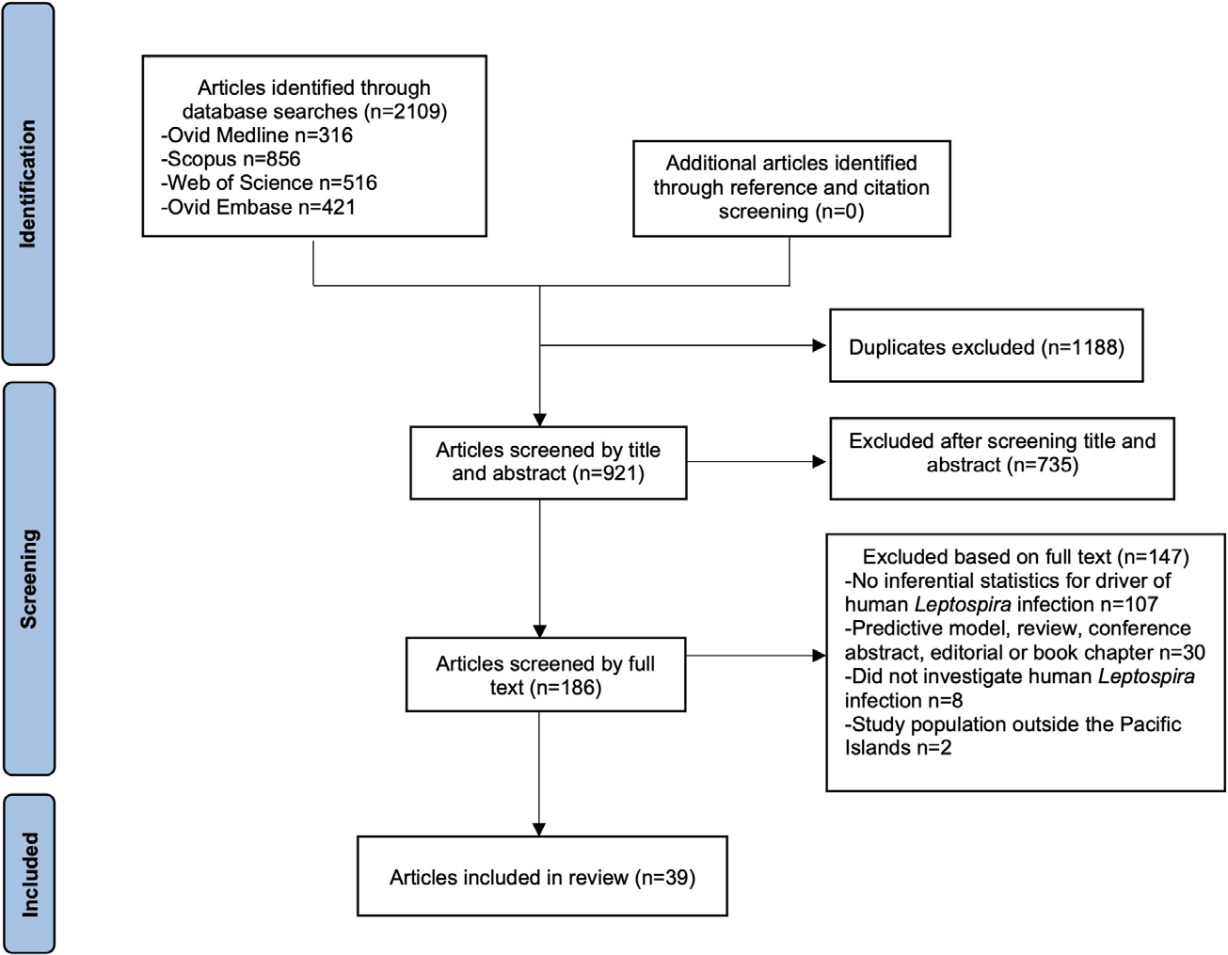
PRISMA flow diagram [8] for the article selection process.

There were 39 articles included in this review (see Figure 1). One of these articles reported four distinct studies on leptospirosis outbreaks between 1985 and 1986 in New Caledonia [14], and hence, a total of 42 studies were included. Reference and citation screening of included articles and relevant excluded reviews yielded no further eligible articles.

### Study characteristics

Of the 42 included studies, the most common study design was cross-sectional (*n* = 17, 40%) followed by case series (*n* = 14, 33%) and test-negative case–control designs (*n* = 7, 17%), all of which were classified as level IV on the NHMRC evidence hierarchy. Three articles (7%) reported prospective cohort studies (level II) [15, 16, 30], and the remaining study was an ecological study (level IV) [17] (see Table 2).

**Table 2.** Study location, period, design, and population and sample characteristics of included studies

References	Study location	Reported study period	Study design (NHMRC level)	Study population	Sample size	Gender/sex distribution	Age distribution
[14] (Study 1)^a^	New Caledonia (Nera)	January 1985–December 1986	Case series (IV)	General population	Total population 3,410 60 cases	Total: male 1851 (54.3%) Cases: male 40, female 20	Cases: <20: 1 (2%); 20–29: 10 (17%), 30–39: 14 (23%), 40–49: 16 (27%), 50–59: 11 (18%), >59: 8 (13%)
[14] (Study 2)^a^	New Caledonia (Nera)	January 1985–December 1986	Cross-sectional (IV)	General population	41 sampled 16 seropositive	Total: male 7,487 (51.2%) Cases: male 21, female 5	Cases: <20: 0, 20–29: 5 (19%), 30–39: 6 (23%), 40–49: 7 (27%), 50–59: 5 (19%), >59: 3 (12%)
[14] (Study 3)^a^	New Caledonia (Coulée)	January 1985–December 1986	Case series (IV)	General population	Total population 14,614 26 cases	Total: male 7,487 (51.2%) Cases: male 21, female 5	Cases: <20: 0, 20–29: 5 (19%), 30–39: 6 (23%), 40–49: 7 (27%), 50–59: 5 (19%), >59: 3 (12%)
[14] (Study 4)^a^	New Caledonia (Coulée)	January 1985–December 1986	Cross-sectional (IV)	General population	93 sampled 16 seropositive	NR	NR
[15]^b^	New Zealand	February 2008–May 2011	Prospective cohort (II)	Abattoir workers	592 participants 51 new infections	Total: male 256 (66.7%), female 128 (33.3%)	Total: <=40: 99 (25.8%), 40–50: 96 (25.0%), 50–57.5: 93 (24.2%), >57.5: 96 (25.0%)
[16]^b^	New Zealand	February 2008–May 2011	Prospective cohort (II)	Sheep abattoir workers	567 participants, 384 for follow–up (cases NR)	Male 256 (66.7%), female 128 (33.3%)	NR
[17]^c^	New Zealand	Not reported	Ecological (IV)	Dairy farm workers	25 cases farms, 27 control farms (number of individuals NR)	NR	NR
[18]	Hawaii	1970–1984	Case series (IV)	General population	Total population NR 195 cases	Male 80.6%	NR
[19]	New Zealand	February–March 2008	Cross-sectional (IV)	Slaughterhouse workers	242 sampled 23 seropositive	Male 145, female 97	Age terciles in population: 20–40, 41–47, > = 48 Median age (seropositive) 54 years (IQR: 47–59) Median age (seronegative) 48 years (IQR: 35–56)
[20]	Palau, Guam, Federated States of Micronesia, Vanuatu, Fiji, Tonga, Wallis, Futuna, and French Polynesia	September 2003–December 2005	Test-negative case–control (IV)	Hospital patients	263 suspected 69 confirmed cases	Male: female = 3:1	Median age (suspected cases) 30 years (range 1–70) Median age (confirmed cases) 27 years (range 7–67)
[21]	New Zealand	July–November 1978	Cross-sectional (IV)	Meat inspectors	1,003 sampled 103 seropositive	NR	NR
[22]	New Zealand	1979–1980	Cross-sectional (IV)	Meat inspectors and workers	Inspectors: 1215 sampled, 121 seropositive Workers: 1248 sampled, 77 seropositive	Meat workers: female 94 NR for meat inspectors	NR
[23]	New Zealand	Not reported	Cross-sectional (IV)	Dairy farm workers	308 sampled 137 seropositive	NR	NR
[24]	French Polynesia	January 2007–December 2017	Case series (IV)	General population	Total population 280,000 1,365 cases (851 confirmed, 505 probable)	Cases: male 1,032 (76.1%), female 324 (23.9%) (sex ratio 3.2)	Men: average age 33.7 years +/− 16.2 (range 2–80) Female: average age 35.2 years +/− 17.8 (range 1–88)
[25]^a^	New Caledonia	January 1985–December 1986	Case series (IV)	General population	Total population 145,368 193 cases	Total: 51.5% of males Cases: 137 male, 56 female	Cases: <20: 5 (2.9%), 20–29: 48 (24.8%), 30–39: 43 (22.2%), 40–49: 59 (30.5%), 50–59: 27 (13.9%), >59: 11 (5.7%)
[26]	Federated States of Micronesia	June–September 2011	Test-negative case–control (IV)	Hospital patients	54 tested 11 confirmed cases	Male 33 (case frequency = 27.3%)	Age 10–24: *n* = 20 (case frequency = 35.0%)
[27]	French Polynesia	March 2004–March 2005	Test-negative case–control (IV)	Hospital patients	113 participants 33 cases (22 confirmed, 11 probable)	Male 82 (72.6%)	Mean 30.5 years (median 30, range: 1–69)
[28]	New Zealand	January 2004–December 2010	Case series (IV)	General population	Total population NR 97 cases (86 confirmed, 8 probable, 3 pending)	NR	Cases: median 41 years (range 17–72)
[29]^b^	New Zealand	November 2009–March 2010	Cross-sectional (IV)	Abattoir workers	567 participants 62 seropositive	Total: deer workers: 9 female; beef workers: 45 female; NR for sheep workers	NR
[30]^b^	New Zealand	Not reported	Prospective cohort (II)	Abattoir workers	384 sampled 49 seropositive	Total: male 256 (66.7%), female 128 (33.3%)	Total: <=40: 99 (25.8%), 40–50: 96 (25.0%), 50–57.5: 93 (24.2%), >57.5: 96 (25.0%)
[31]	New Zealand	2010–2015	Case series (IV)	General population	Total population NR 442 cases	Cases: male 398, female 44 Average incidence rate (cases per year): male 3, female 0.3	Age 20–59 years: 83.5% of cases Average incidence rate (cases per year): 20–29: 2.1, 30–39: 2.3, 40–49: 2.8, 50–59: 3
[32]	New Caledonia	January–June 2008	Test-negative case–control (IV)	Hospital patients	135 cases (101 confirmed, 34 probable) For inferential statistics: 98 cases, 410 controls	Cases: male/female = 2.2	Cases: mean age 35.2 years (range 4.6–84.3)
[33]	Hawaii	January 1974–December 1998	Case series (IV)	General population	Total population NR 709 cases (353 confirmed, 180 probable, 176 suspected)	Cases: male 92%	Cases: median age 33 years (range 1–78)
[34]	New Caledonia	January 2006–December 2016	Case series (IV)	General population	Total population 268,767 904 cases (700 confirmed, 204 probable)	Cases: 604 male, 300 female (M/F = 2.01)	Cases: median age 33.6 years (range 1.1–84.8)
[35]^d^	American Samoa	May–July 2010	Cross-sectional (IV)	General population	807 participants 125 seropositive	As per reference [36]	As per reference [36]
[36]^d^	American Samoa	May–July 2010	Cross-sectional (IV)	General population	807 participants 125 seropositive	Total: male 423 (52%) Seropositive: male 93 (74%)	Total: mean 40 years (range 17–87)
[37]^d^	American Samoa	May–July 2010	Cross-sectional (IV)	General population	807 participants 125 seropositive	As per reference [36]	As per reference [36]
[38]^e^	Fiji	September–December 2013	Cross-sectional (IV)	General population	2,152 participants 417 seropositive	Total: male 985 (45.8%), female 1,160 Seropositive: 234 male, 182 female	Total: mean 33.6 years (range 1–90, standard deviation 19.8)
[39]	Hawaii	April–March 2002	Cross-sectional (IV)	Army blood bank donors	488 subjects 7 seropositive	Total: male 189 (79.7%) Seropositive: male 3 (42.9%)	Age 18–30: total 292 (59.8%), seropositive 7 (100%)
[40]	New Zealand	NR	Cross-sectional (IV)	Dairy farm workers	226 participants, 213 sampled 84 seropositive	Total (213 sampled): male 174 (81.7%) female 39 (18.3%)	NR
[41]	Wallis and Futuna	January 2008–June 2015	Test-negative case–control (IV)	Hospital patients	338 suspected, 165 confirmed, 173 excluded cases	Total: male 251, female 87 Cases: male 141, female 24	Males aged 10–30 years: 83 Females aged 40–59 years: 23
[42]	Futuna	2004–2014	Case series (IV)	General population	Total population 3,612 382 cases (confirmed/probable NR)	Male: female = 5:4	Peak incidence in 20–29 years age class
[43]^e^	Fiji	2013	Cross-sectional (IV)	General population	2046 participants (number seropositive NR)	NR	NR
[44]	Vanuatu	January 2013–August 2014	Test-negative case–control (IV)	Hospital patients	161 patients 12 cases (5 confirmed, 7 probable) 29 seropositive	Male 77, female 84 (male: female =0.92)	All patients >15 years Median age 30 years Mean age 34 years (IQR 23–40, range 15–75)
[45]	New Caledonia	1989	Case series (IV)	General population	Total population 165,000 144 cases	Cases: male 100, female 44	Cases (out of 126 cases): median age 35 years (range 3–104)
[46]	New Zealand	May–October 2013	Cross-sectional (IV)	Beef, sheep, and deer farmers	178 participants 12 seropositive	Total: male 159 Seropositive: male 12	Total: mean age 53 years (minimum 20, maximum 76)
[47]	New Zealand	May/June 2012	Cross-sectional (IV)	Veterinarians	277 participants 14 seropositive	Female 109 (39%)	Median 42 years (minimum 22, quartile 1 33, quartile 3 53, maximum 73)
[48]	Hawaii	July 1988–June 1989 (Big Island); July–December 1988 (Kauai)	Test-negative case–control (IV)	Hospital patients	Big Island: 172 participants, 123 followed, 20 seropositive Kauai: 100 participants, 59 followed, 13 cases Inferential statistics: 33 cases, 77 controls	Case–control participants: 96% of males	Total: median 36 years (range 26–71) Case–control participants: mean 37 years (standard deviation 12.1)
[49]	New Zealand	Not reported	Cross-sectional (IV)	Pig farmers	70 participants 20 seropositive	Seropositive: 27% of males, 8% of females	NR
[50]	Palau	May 2000–June 2006	Case series (IV)	General population	Total population 20,000 81 cases	Cases: male 59 (72.8%)	Cases: mean 31.8 years, median 31 years (range 1.5–76 years)
[51]	New Zealand	1990–1998	Case series (IV)	General population	Total population NR 1,397 cases	Cases: male 1,238 (90.4%)	Median 36 years (range 4–80)
[52]	Fiji	December 2011–May 2012	Case series (IV)	People who sought medical care	Total population 340,000 1,217 suspected cases (31 confirmed, 283 probable)	Suspected cases: male 61%	Suspected cases: median 30 years (IQR: 19–42)

Abbreviations: IQR, interquartile range; NHMRC, National Health and Medical Research Council; NR, not reported.

a Reported the same study period from 1985 to 1986 in New Caledonia.

b Reported the same prospective cohort study conducted in New Zealand from February 2008 to May 2011. Reference [29] reported one sample undertaken between November 2009 and March 2010.

c Study design was reported by the authors as case–control; however, this referred to the farms and not the individuals measured. For this review, the study design was classified as ecological as the exposures were measured after the outcome.

d Reported on the same seroprevalence study conducted in American Samoa in 2010.

e Reported on the same seroprevalence study conducted in Fiji in 2013.

In terms of geographic distribution, 16 studies (38%) were conducted in New Zealand and 24 studies (57%) represented other Pacific Islands, including New Caledonia (*n* = 8, 19%), Hawaii (*n* = 4, 10%), American Samoa (*n* = 3, 7%), Fiji (*n* = 3, 7%), French Polynesia (*n* = 2, 5%), Wallis/Futuna (*n* = 2, 5%), Palau (*n* = 1, 2%), Vanuatu (*n* = 1, 2%), and Federated States of Micronesia (*n* = 1, 2%), and the final study examined several Pacific Islands [20].

With respect to study outcomes, there were 23 studies (55%) reporting on human leptospirosis incidence, 18 seroprevalence surveys (43%), and two studies (5%) investigating both incidence and seroprevalence. Approximately half of the studies (48%) used a combination of direct diagnostic methods, such as polymerase chain reaction (PCR), culture and dark field microscopy, and indirect methods, such as microscopic agglutination test (MAT) and enzyme-linked immunosorbent assay (ELISA), to investigate *Leptospira* infection. There were 21 studies (50%) that used MAT for diagnosis, often with different antibody titre cut-offs, and the remaining study did not specify the diagnostic method used [50] (see Table 3).

**Table 3.** Leptospirosis outcomes, diagnostic tests, serogroups detected, and drivers investigated in included studies

References	Leptospirosis outcome(s)	How outcome(s) were measured	Serogroups	Method of serogroup identification	Drivers of infection investigated using inferential statistics
[14] (Study 1)	Incidence	Direct: dark field microscopy; indirect: ELISA and agglutination test for 14 serotypes	*L. icterohaemorrhagiae* 33 (55%), *L. pomona* 7 (12%), *L. cynopteri* 4 (7%), *L. canicola* 3 (5%), *L. grippotyphosa* 3 (5%), *L. tarassovi* 3 (5%), *L. hardjo* 2 (3%), *L. autumnalis* 2 (3%), *L. bataviae* 2 (3%), *L. ballum* 1 (2%)	Agglutination–lysis reaction against serotypes	**Age; sex**; ethnicity; **location**
[14] (Study 2)	Seroprevalence	Direct: dark field microscopy; indirect: ELISA and agglutination test for 14 serotypes	7 different serotypes identified: *L. icterohaemorrhagiae* 17 (65%)	Agglutination–lysis reaction against serotypes	**Freshwater contact**
[14] (Study 3)	Incidence	Direct: dark field microscopy; indirect: ELISA and agglutination test for 14 serotypes	7 different serotypes identified: *L. icterohaemorrhagiae* 17 (65%)	Agglutination–lysis reaction against serotypes	**Age; sex**; ethnicity; **location**
[14] (Study 4)	Seroprevalence	Direct: dark field microscopy; indirect: ELISA and agglutination test for 14 serotypes	*L. icterohaemorrhagiae* 8	Agglutination test	**Freshwater contact; murid contact**; occupation
[15]	Incidence	MAT for Pomona and Hardjo-bovis at doubling dilutions from 1:24 to 1:1536; suspected cases > = 1:48 titre	*L. borgpetersenii* serovar Hardjo = 2.3% (95% CI 1.4–4.0) cumulative annual risk of infection, *L. interrogans* serovar Pomona = 5.8% (95% CI 4.2–8.0) cumulative annual risk of infection	MAT titre	Age; gender; ethnicity; **type of meat work**
[16]	Incidence	MAT titre > = 1:48 or anamnestic response (increase by 2 or more dilutions)	Only Pomona investigated	NA	Age; gender; **work position**; **abattoir plant number;** time worked in occupation; hunting; home slaughter of animals; recreational farming; personal protective equipment
[17]	Seroprevalence	High–risk herds: milkers had titres > = 1:96; controls: milkers with no detectable agglutinin titres at a minimum serum dilution of 1:20	NR	NR	**Leptospirosis in herd**; herd size
[18]	Incidence	Macroscopic slide agglutination test and MAT; cultures of urine, blood, and kidney specimens using Ellinghausen–McCullough–Johnson–Harris media; cases: clinical manifestations and supporting lab findings; presumptive: clinical findings confirmed with serology, culture, or combination of these	Total 186: Icterohaemorrhagiae 83 (44.6%), Canicola 7 (3.8%), Australis 7 (3.8%), Hebdomadis 6 (3.2%), Ballum 4 (2.2%), Pomona (2.2%), Autumnalis 3 (1.6%), Pyrogenes 2 (1.1%), Bataviae 2 (1.1%), Javanica 1 (0.5%), undetermined 67 (36.0%)	Highest MAT titre	Seasonality
[19]	Seroprevalence	MAT with a titre cut-off > = 1:24 for serovars Pomona and Hardjo	Hardjo 10 (4.1%) (titres 1:24–1:192), Pomona 13 (5.4%) (titres 1:24–1:768), both 1	MAT highest titre	**Age; gender**
[20]	Incidence (confirmed cases only)	Confirmed: PCR-positive or fourfold increase in MAT titre; probable = single sample with positive MAT	Total (regional) 69: Australis 19 (28%), Autumnalis 2 (3%), Ballum 2 (3%), Canicola 5 (7%), Icterohaemorrhagiae 31 (45%), Panama 1 (<1%), undetermined 7 (10%), coagglutinated 2 (3%)	MAT highest titre	Age; **gender**; animal contact; hunting; fishing; bathing in freshwater
[21]	Seroprevalence	MAT with an initial serum dilution of 1:24	Pomona 78 (68%), Tarassovi 19 (17%), Hardjo 12 (10%), Copenhageni 4 (4%), Ballum 1 (1%)	MAT titre	**Location; type of meat work**
[22]	Seroprevalence	MAT with an initial serum dilution of 1:24	Meat inspectors: Pomona 90 (68.2%, including 10 concurrent reactions with Tarassovi 6, Hardjo 3, or Copenhageni 2), Tarassovi 19 (14.4%), Hardjo 15 (11.3%), Copenhageni 5 (3.8%), Ballum 3 (0.4%) Meat workers: Hardjo 17, Pomona 40, Tarassovi 2, Copenhageni 21, Ballum 7, 10 dual reactions (Pomona/Hardjo 4, Pomona/Copenhageni 2, Pomona/Tarassovi 1, Ballum/Copenhageni 2, Ballum/Hardjo 1)	MAT titre	**Ethnicity; type of meat work; work position; pig contact outside work**; smoking
[23]	Seroprevalence	Sera examined for leptospiral agglutinins at titres > = 1:24 for the six serovars representing all serogroups known to be endemic in New Zealand	Total (including 54 concurrent reactions) 151: Hardjo 94, Pomona 79, Tarassovi 8, Ballum 5, Copenhageni 11, Australis 7 Reaction to specific serovars only: Hardjo 56, Pomona 41, Tarassovi: 2, Ballum: 3, Copenhageni: 1, Australis 1	Positive MAT titre > = 1:24 for serovar	**Sex; leptospirosis in herd; herd size; milking of animals; vaccination of herd**; smoking; personal protective equipment; **type of shed**; buying in stock; animals on farm; docking cow tails; milk for factory versus town supply
[24]	Incidence (annual incidence rates using confirmed and probable cases)	Confirmed: symptoms with positive culture, PCR confirmation or IgM seroconversion; probable: symptoms and IgM detection ELISA on a single sample collected more than 1 week after onset of symptoms; excluded: symptoms with PCR-negative or ELISA IgM-negative	NR	NA	**Sex; location**
[25]	Incidence, seroprevalence	Direct diagnosis with culture; indirect diagnosis with ELISA	Cases: *L. icterohaemorrhagiae* 116 cases (59.8%), *L. pomona* 16 (8.3%). Close contacts: *L. icterohaemorrhagiae* 40 (63.5%), *L. hardjo* (11.1%) At-risk cases: *L. icterohaemorrhagiae* 57 (45.9%), *L. pomona* 10, *L. hardjo* 10	Agglutination–lysis reaction against serotypes	**Age; sex**; ethnicity; **occupation; location**
[26]	Incidence (confirmed cases only)	Positive MAT result = titre of 1:400 or greater on a single specimen or fourfold increase between acute and convalescent specimens	Pomona 1, Celledoni 5, Copenhageni 10 (five confirmed cases), Australis 4, Canicola 3, Hebdomadis 2, Autumnalis 2, Cynopteri 4, Ballum 2 (1 confirmed case), Djasiman 2, Panama 1, LT751 12 (five confirmed cases)	20 serovar MAT panel: titre >1:50	Age; sex; **occupation**; education level; drinking from stream; bathing from stream; pigs, dogs or rats around the home; swimming or standing in freshwater; walking through mud; skin wounds; pig slaughter; **tending to garden or crops**
[27]	Incidence (confirmed or probable)	Suspected cases: meet World Health Organization clinical criteria; confirmed: positive PCR or positive seroconversion ELISA; probable: positive IgM on ELISA and/or MAT titre >1:200	Raiatea (total 18): Australis 6, Autumnalis 1, Icterohaemorrhagiae 11 Nuku Hiva (total 11): Australis 1, Ballum 2, Canicola 7, Icterohaemorrhagiae 1	MAT	Location; fishing; **swimming in river; hunting**; contact with rats, dogs, pigs or horses
[28]	Incidence (confirmed or probable or pending)	Confirmed: clinical symptoms and either > = 400 titre on MAT or fourfold increase in titre between two consecutive samples; probable: clinical symptoms and single raised MAT of > = 400	Australis 1 (1%), Canicola 1 (1%), Copenhageni 3 (3.1%), Tarassovi 14 (14.4%), Ballum 16 (16.5%), Pomona 23 (23.7%), Hardjo–bovis 24 (24.7%), not identified 15 (15.5%)	MAT	**Seasonality**
[29]	Seroprevalence	MAT for Pomona and Hardjo-bovis at doubling dilutions from 1:24 to 1:1536; suspected cases > = 1:48 titre	Seroprevalence: either Pomona or Hardjo 11% (95% CI 8–14), Pomona 5% (95% CI 3–7), Hardjo 8% (95% CI 6–10)	MAT titre > = 1:48	**Age**; gender; **work position; abattoir plant number; personal protective equipment; time worked in occupation**
[30]	Incidence	MAT for Pomona and Hardjo-bovis at doubling dilutions from 1:24 to 1:1536; suspected cases > = 1:48 titre	NR	MAT titre	Age; gender; ethnicity; **work position**; **abattoir plant number**; personal protective equipment; time worked in current abattoir; **time worked in occupation**; animal urine splashed in face; hunting; recreational farming; home slaughter of animals
[31]	Incidence	MAT, NAT, and/or isolation techniques	Borgpetersenii sv. Hardjo 110, Borgpetersenii sv. Ballum 85, Borgpetersenii sv. Tarassovi 30, Interrogans sv. Pomona 69, Interrogans sv. Canicola 8, Interrogans sv. Copenhageni 12, Interrogans serogroup Australis 2, Kirschneri serogroup Grippotyphosa 2, 1 co-infection with Hardjo and Pomona, 123 no serovar identified	NR	**Gender**; location
[32]	Incidence (confirmed or probable)	Molecular diagnosis: real-time PCR with sera or urine; serological diagnosis: MAT; confirmed: positive PCR or seroconversion in paired samples; probable: clinical presentation and single MAT titre > = 800	Ballum 2, Tarassovi 20, Australis 14, Icterohaemorrhagiae or Copenhageni 32, Panama 1, Pomona 1, Pyrogenes 13, unknown (PCR-diagnosed) or co-agglutinins 70	Molecular testing; MAT	**Freshwater swimming; fishing; hunting; contact with any animal, cattle, pigs, horses**, dogs, or **rodents**
[33]	Incidence (confirmed only)	Confirmed: clinically compatible illness with fourfold or greater increase in MAT titre, isolation of Leptospira from specimen, or demonstration of Leptospira in the specimen by immunofluorescence	Culture isolates (81): Australis 19, Ballum 5, Bataviae 5, Icterohaemorrhagiae 43, Pomona 1, Sejroe 2 Isolate or presumptively (324): Andamana 1, Australis 67, Autumnalis 8, Ballum 22, Bataviae 7, Canicola 18, Cynopteri 1, Djasiman 1, Hebdomadis 1, Icterohaemorrhagiae 170, Mini 6, Pomona 2, Pyrogenes 4, Sejroe 13, Tarassovi 3, identification pending 6	Definitive from culture isolates; presumptive from MAT highest titre	**Age; ethnicity; location**
[34]	Incidence (confirmed or probable)	PCR from serum or urine (targeting lfb1 from 2006 to 2009, lipL32 2010 onwards), serological MAT; confirmed: positive qPCR or seroconversion; probable: compatible clinical presentation; and a single MAT titre > = 800	Total (743): Icterohaemorrhagiae 426 (57.3%), Pyrogenes 148 (19.9%), Australis 86 (11.6%), Ballum 47 (6.3%), Pomona 12 (1.6%) Suggested by MAT but not confirmed by PCR/isolation: Panama 13, Canicola 8, Autumnalis 1, Tarassovi 1	Highest MAT titre (385 cases); genotyping using diagnostic PCR (449 cases)	**Age; seasonality**
[35]	Seroprevalence	As per [36]	*L. interrogans* serovar LT751: participants 13, seropositive 13, RR 16.24 (*P* = 0.00032) *L. interrogans* serovar LT1163: participants 130, seropositive 14, RR 5.94 (*P* = 0.02)	As per [36]	**Gender; indoor-versus-outdoor occupation; heard of leptospirosis; house altitude; vegetation type; soil type; proximity of house to piggeries**
[36]	Seroprevalence	MAT with 23 serovars; positive: MAT titre > = 1:50	*L. interrogans* serovars Hebdomadis 48.3%, LT751 25.5%, LT1163 17.4%	Highest MAT titre	**Gender; indoor-versus-outdoor occupation; household income; heard of leptospirosis; swimming at beach; swimming or walking in rain puddles; fishing; house altitude; proximity of house to piggeries**
[37]	Seroprevalence	Used a MAT panel of 23 serovars; MAT titre > = 1:50	Serovar Hebdomadis (serogroup Hebdomadis) 72 (48.3%), serovar LT751 (serogroup Australis) 38 (25.5%), serovar LT1163 (serogroup Pyrogenes) 26 (17.4%)	MAT	**Location; population density; vegetation type**
[38]	Seroprevalence	MAT dilutions from 1:50 to 1:3200 (Pohnpei, Australia, Canicola, Copenhageni, Hardjo, Ballum); seropositive: MAT titre > = 1:50	*L. interrogans* serovars Pohnpei, Australis, Canicola, Copenhageni, and Hardjo; and *L. borgpetersenii* serovar Ballum accounted for 86.7% of reactive tests Pohnpei: 351 (84.2%, 95% CI 80.3–87.5)	Highest MAT titre	**Gender; ethnicity; metered water available at home; indoor-versus-outdoor occupation; urban versus rural; community poverty rate; proximity of home to water body; exposure to rodents, mongooses, pigs, cows, goats, horses, chickens, cats, or dogs; total cattle density; rainfall**
[39]	Seroprevalence	MAT for leptospiral antibodies with 16 serovars; screened at dilution of 1/100 against Leptospira	Total (7): Bratislava 5, Australis 1, Autumnalis 1	Highest reactive MAT dilution	**Age; gender**
[40]	Seroprevalence	Serum tested for antibodies to *L. interrogans* serovars Hardjo, Pomona, Ballum, Copenhageni, and Tarassovi using MAT with minimum final dilution 1:24.	Hardjo 48 (57.1%), Pomona 29 (34.5%), Copenhageni 4 (4.8%), Ballum 3 (3.6%), Tarassovi 0, 14 seropositive to 2 or more serovars; concurrent Hardjo/Pomona titres 12 (5.6%)	MAT titre	**Sex; milking of animals; personal protective equipment**; milk for factory versus town supply; shed type; leptospirosis in herd; **pig keeping**; udder washing or teat stimulation
[41]	Incidence (suspected or confirmed)	IgM and ELISA and/or seroconversion with MAT and/or PCR; biologically confirmed by seroconversion or PCR	NR	NR	**Age; location**
[42]	Incidence (confirmed or probable)	2004–2007: MAT 2008–2014: IgM ELISA with MAT, PCR; confirmed: current or recent fever, positive real–time PCR, seroconversion from nil to >400 in MAT or twofold increase in IgM or fourfold in MAT; probable: recent or current fever and single IgM ELISA of 15 units or single MAT > = 800	NR	NA	**Location; rainfall**
[43]	Seroprevalence	Blood sample MAT	NR	NA	**Community poverty rate; rural residential setting; rainfall; distance to river; total cattle density**
[44]	Incidence (confirmed or probable cases), seroprevalence	PCR, 24 panel MAT, ELISA, urine samples; probable acute infection: MAT > = 800 or ELISA > = 18; confirmed acute: positive qPCR	Total (15): Pyrogenes 1, Icterohaemorrhagiae 1, Louisiana 1, Panama 1, Australis 8, 3 co-agglutinations	Highest MAT titre	Sex; location; home water source; occupation; fishing; contact with surface water; hunting; contact with pigs, dogs, cats, horses, goat, sheep or cow; rodents seen in vicinity
[45]	Incidence	MAT titre greater than or equal to 1:100 using 23 live antigens representing all pathogenic serogroups of *L. interrogans*, or strains isolated from blood, urine, or cerebrospinal fluid	Icterohaemorrhagiae 59, Tarassovi 20, Sejroe 12, Canicola 11, Pyrogenes 9, Pomona 9, Panama 3, Djasiman 2, Cynopteri 2, Mini 1, Ballum 1, Australis 1, cross–reactive 13, undetermined 1	MAT	**Gender**; fishing; hunting; swimming; animal contact
[46]	Seroprevalence	Modified MAT for Hardjo, Pomona, Ballum, Copenhageni, and Tarassovi with twofold dilutions (1:24 to 1:3072)	Pomona 5 (2.6%), Ballum 4 (2.1%), Copenhageni 2 (1.0%), Tarassovi 1 (0.4%), Hardjo–bovis 0	MAT; last dilution able to agglutinate more than 50% of leptospires was taken as final titre for each serovar	Age; location; smoking; **assisting calving, fawning,** or lambing; pet dog or cat in house; exposure to dairy cattle or **goats**; home slaughter of animals; **wild deer, possum, rabbit**, hedgehog, mice, wild pig, rat, or wild cat abundance on farm; **species being farmed**; **flat terrain of farm**; valley pond water source; hay fever; flooding of farmland; farm location; farm effluent into oxidation ponds versus pastures; leptospirosis in herd; hunting; freshwater contact; camping; water sports; fishing; hiking, any animal contact; animal urine contact; shearing of animals; castrating animals; time worked in occupation; heard of leptospirosis; milking cows; smoking
[47]	Seroprevalence	Modified MAT with Hardjo, Pomona, Ballum, Copenhageni, and Tarassovi. Twofold dilutions (1:24 to 1:3072)	Pomona 7 (2.5%, 95% CI 1.0–5.1), Hardjo 6 (2.2%, 95% CI 0.8–4.7), Ballum 1 (0.4%, 95% CI 0.0–2.0), Copenhageni 1 (0.4%, 95% CI 0.0–2.0), Tarassovi 0 (0.0%, 95% CI 0.1–2.0)	Highest MAT titre with last dilution to agglutinate >50% of leptospires	Gender; dairy or dog–cat exposure; hunting; camping lakes/rivers; fishing; owning deer, pigs, cattle, sheep, horses, dogs, or cats; time spent with animals; **home slaughter of animals**
[48]	Incidence	Confirmed: leptospires isolated from blood cultures or fourfold increase in MAT titre or leptospires demonstrated in tissue samples by direct fluorescence antibody testing; presumptive: minimum MAT titre of 1:200 but not fourfold increase	Total (20): Icterohaemorrhagiae 10 (50%), Ballum 3 (15%), Canicola 2 (10%), Hebdomadis 1 (5%); Pomona 1 (5%), Australis 1 (5%), unknown 2 (10%)	MAT titre	Age; sex; location; seasonality; **household water catchment system**; drinking surface water (stream); **skin wounds; handling animal tissues**; rainfall
[49]	Seroprevalence	Samples were tested by MAT with an initial serum dilution of 1:24 using cultures of Australis, Ballum, Copenhageni, Hardjo, Pomona, and Tarassovi as antigens	Pomona 15 (23%), Hardjo 4 (6%), Tarassovi 2 (3%), Ballum 1 (1.5%), Copenhageni 0, Australis 0 (includes two dual reactions: Hardjo/Pomona, Hardjo/Tarassovi)	MAT	Gender; personal protective equipment use; part-time-versus-full-time work; time worked in occupation; slaughtering of animals at work; buying in stock; leptospirosis in herd; vaccination of herd; farm effluent into oxidation ponds versus pastures; smoking; number of breeding sows and fattening pigs; stock housing
[50]	Incidence	Notified cases	NR	NR	Urban versus rural
[51]	Incidence	Single serological titre > = 400 on MAT, a greater than fourfold rise in titres between two sequential specimens or isolation of leptospires from clinical specimens	Total (1266): *L. borgpetersenii* sv. Hardjo 584 (46.1%), *L. interrogans* Pomona 309 (24.4%), *L. borgpetersenii* sv. Ballum 151 (11.9%), *L. interrogans* sv. Bratislava 58, *L. borgpetersenii* sv. Tarrassovi 54, *L. interrogans* sv. Copenhageni 52, *L. interrogans* sv. Canicola 11, *L. interrogans* sv. Australis 3	NR	**Gender; occupation; ratio of dairy cattle numbers to human population**
[52]	Incidence (confirmed or probable)	ELISA, PCR, and MAT using a panel of 28 *Leptospira* serovars; confirmed: positive quantitative PCR, MAT titre > = 1:800, or immunohistochemical detection of leptospira in tissues; probable: unconfirmed cases with positive anti–leptospira IgM ELISA or MAT titre 1:200–1:400	Total (17): Icterohaemorrhagiae 7, Ballum 3, Australis 4, Pomona 3	Genotyping	**Flooding**

Abbreviations: CI, confidence interval; ELISA, enzyme-linked immunosorbent assay; MAT, microscopic agglutination test; NA, not applicable; NR, not reported; PCR, polymerase chain reaction.

*Note*: statistically significant drivers of infection (*P* < 0.05 or if no *P*-value reported, significance as reported by authors) presented in bold. If unadjusted and adjusted estimates were reported, the driver of infection is bolded if adjusted estimates were significant (refer to Supplementary Material for further details).

### Risk of bias within studies

As described previously, 39 studies (93%) were classified as level IV, the lowest level of evidence on the NHMRC evidence hierarchy.

**Figure 2.**
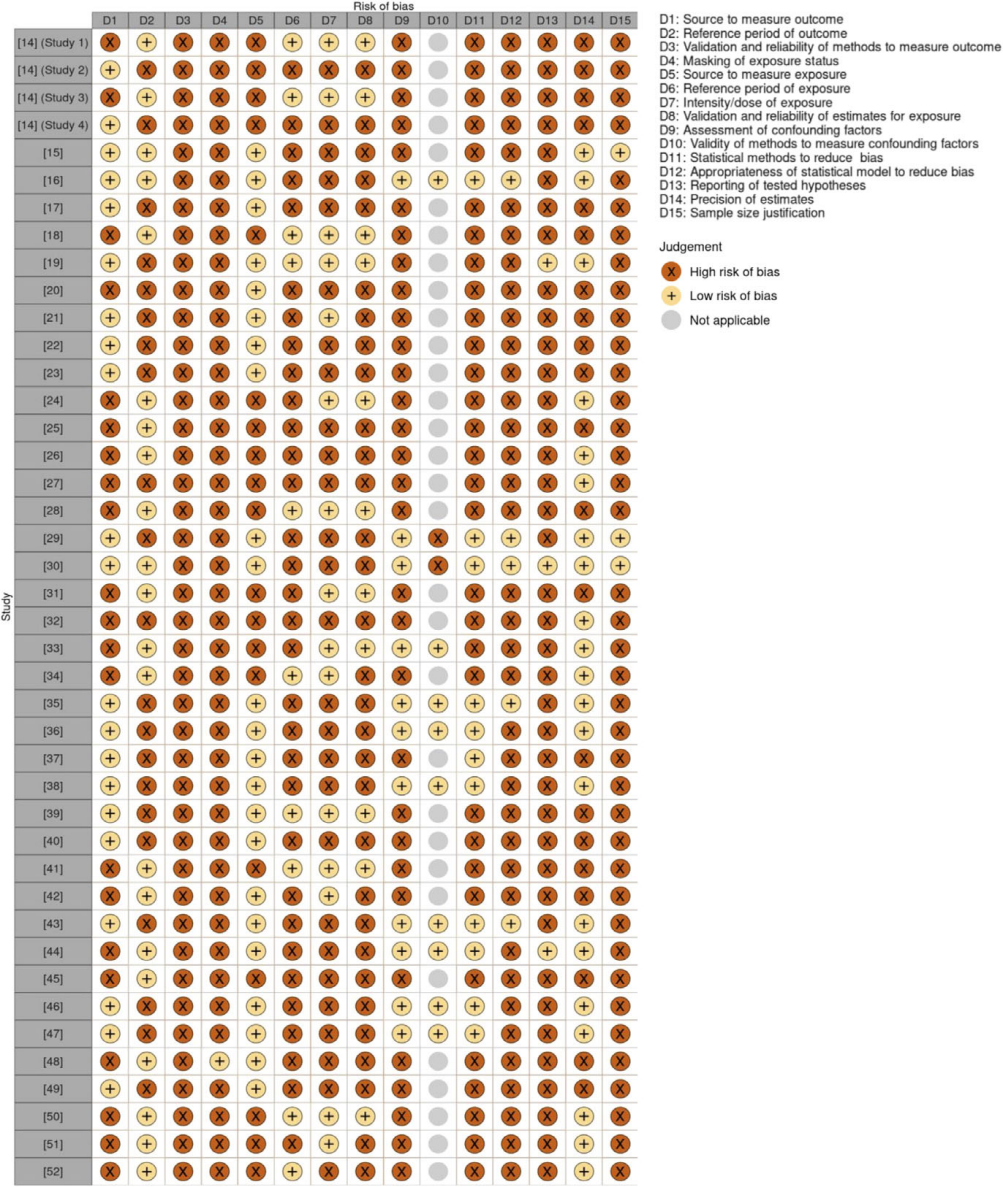
Traffic light plot representing study-by-study potential biases for each domain of the Methodological Evaluation of Observational Research Checklist (MEVORECH) tool [12] using the risk-of-bias visualization tool ‘robvis’ [53]. *Note*: 31 studies were considered not applicable for D10 as they did not assess for confounding factors.

Using the MEVORECH tool, potential biases were identified for all studies across several domains (see Figure 2). The authors did not state the validity and reliability of their methods to measure human *Leptospira* infection and/or the drivers of infection and often used medical records to sample data rather than methods designed specifically for the purpose of their study. Several studies additionally included probable cases identified from a single positive ELISA and/or MAT result (see Table 3). Furthermore, some studies did not assess the duration and frequency of exposure to drivers of infection, primarily for occupational and lifestyle factors. Lastly, many studies did not account for confounding factors and some failed to provide an effect size with confidence intervals for their estimates.

### Synthesis of results

#### Sociodemographic drivers

There were 31 studies (31/42, 74%) that reported on sociodemographic drivers of infection including gender/sex (*n* = 24, 57%), age (*n* = 15, 36%), ethnicity (*n* = 7, 17%), poverty (*n* = 3, 7%), household water supply (*n* = 3, 7%), awareness of leptospirosis (*n* = 3, 7%), and education level (*n* = 1, 2%) (see Supplementary Table S2 for details).

Males were associated with a significantly higher risk of infection compared to females in most studies where gender/sex was investigated (14/24, 58%). One study of army blood bank donors in Hawaii reported higher seroprevalence in females [39], and other studies found no association with gender/sex in occupational settings [15, 16, 29, 30, 47, 49]. Older individuals, particularly those aged between 20 and 60 years, had a higher infection risk in several studies (6/15, 40%), including in New Zealand [19, 29], New Caledonia [14, 25, 34], and Hawaii [33]. However, in Futuna [41] and in a New Caledonia notification data study [34], school-aged children and young adults had the greatest risk of infection. Furthermore, in Fiji [38] and New Zealand [22], there was higher seroprevalence reported in Indigenous populations compared to other ethnicities.

Poverty was another significant driver of infection reported in each of the three studies where it was investigated. The highest seroprevalence was identified in households with an annual income between 20,000 and 30,000 USD in American Samoa [36] and in Fijian communities with a poverty rate greater than 40% [38, 43]. In addition, households with an untreated water supply were associated with a higher risk of infection in Fiji [38], Hawaii [48], and Vanuatu (unadjusted estimates only) [44].

#### Occupational drivers

There were 19 studies (19/42, 45%) that investigated occupational drivers of infection, including meat worker-specific factors (*n* = 6, 14%), farm worker-specific factors (*n* = 6, 14%), personal protective equipment (PPE) (*n* = 6, 14%), time worked within an occupation (*n* = 5, 12%), type of occupation (*n* = 4, 10%), indoor-versus-outdoor occupation (*n* = 3, 7%), animal urine contact (*n* = 2, 5%), and veterinarian-specific factors (*n* = 1, 2%) (see Supplementary Table S3 for details).

In New Zealand, leptospirosis incidence rates were highest in meat and farm workers [51]. In meat workers, high-risk work positions were working on the slaughterfloor, offal removal, and stunning/pelting [16, 22, 29, 30]. Two studies (2/3, 67%) reported increased seroprevalence in pig workers compared to workers processing other meats [21, 22], whereas another study reported higher annual infection risk in sheep abattoir workers than in beef or deer workers [15]. In farm workers, farming deer was associated with significantly higher seroprevalence compared to farming beef or sheep, and farmers assisting in calving or fawning also had a higher risk of infection [46]. Moreover, the primary farm-related characteristic associated with higher seroprevalence in workers was the flat terrain of the farm being greater than 25% [46]. Five studies (5/6, 83%) that investigated PPE use in farmers or meat workers reported no reduction in infection risk, irrespective of the type of PPE. In fact, one study instead found that always or often wearing a facemask or safety glasses increased the odds of seropositivity in meat workers [29].

In American Samoa and Fiji, three studies (3/3, 100%) found that seropositive cases were significantly more likely to be outdoor workers compared to indoor workers [35, 36, 38]. In New Caledonia, one study (1/2, 50%) identified higher seroprevalence in farmers compared to other occupations [25]. Lastly, in the Federated States of Micronesia, those with infection were significantly more likely to be students compared with farmers or other occupations [26].

#### Lifestyle drivers

There were 15 studies (15/42, 36%) that reported on lifestyle drivers of human *Leptospira* infection, including water-associated exposures (*n* = 11, 26%), non-water-based recreational activities (*n* = 10, 24%), smoking (*n* = 3, 7%), and open skin wounds (*n* = 2, 5%) (see Supplementary Table S4 for details).

Swimming was found to be a significant driver of infection in several studies (3/5, 60%), including in New Caledonia [32], American Samoa [36], and French Polynesia [27]. In the Federated States of Micronesia, gardening or tending to crops also increased the risk of infection [26]. In contrast, other recreational activities such as fishing, hunting, and camping were generally not associated with infections [20, 44, 46]. Furthermore, home slaughter of animals was associated with higher seroprevalence in veterinarians [47] but not in meat or farm workers [16, 30, 46].

In addition, one study (1/2, 50%) reported a significant association between skin wounds and *Leptospira* infection [48].

### Environmental drivers

There were 29 studies (29/42, 69%) that reported on environmental drivers of human *Leptospira* infection, including location (*n* = 16, 38%), animal exposures (*n* = 13, 31%), rainfall (*n* = 5, 12%), seasonality (*n* = 4, 10%), geographical characteristics of households (*n* = 4, 10%), vegetation/soil type (*n* = 2, 5%), and human population density (*n* = 1, 2%) (see Supplementary Table S5 for details).

Geographical locations in the Pacific Islands with significantly higher rates of infection included La Nera in New Caledonia [14, 25], Hawaii, and Kauai counties compared to Oahu in Hawaii [33] and the North Island in New Zealand [21]. In American Samoa, serogroup Australis was significantly more common in the Manu’a Islands, whereas serogroup Hebdomadis was only identified in Tutuila [37]. Furthermore, two studies (2/3, 67%), both conducted in Fiji, identified higher seroprevalence in rural settlements compared to urban residences [38, 43].

In terms of animal contact, rodent exposure was a significant factor in several studies (3/6, 50%), including in New Caledonia [14, 32] and Fiji [38]. Exposure to pigs and/or cows was also significantly associated with infections in New Caledonia [32], Fiji [38], and Vanuatu (unadjusted estimates only) [44]. Furthermore, local cattle density was strongly correlated with infections in Fiji [38, 43] and New Zealand [51].

With respect to seasonality – in New Zealand and New Caledonia, there was a lower incidence of leptospirosis during the cool and dry months compared to the warmer months [28, 34]. In Fiji, infections were significantly associated with floods [52] and maximum rainfall in the wettest month [38, 43]. Leptospirosis cases were also strongly correlated with rainfall observed two months earlier in Futuna [42].

Other relevant characteristics of households that were associated with higher seroprevalence included the presence of a water body located less than 100 m from home in Fiji [38, 43], house altitude below the median altitude of the village, households located nearby and below piggeries, agricultural vegetation type, and clay loam soil types in American Samoa [35, 36].

## Discussion

**Figure 3.**
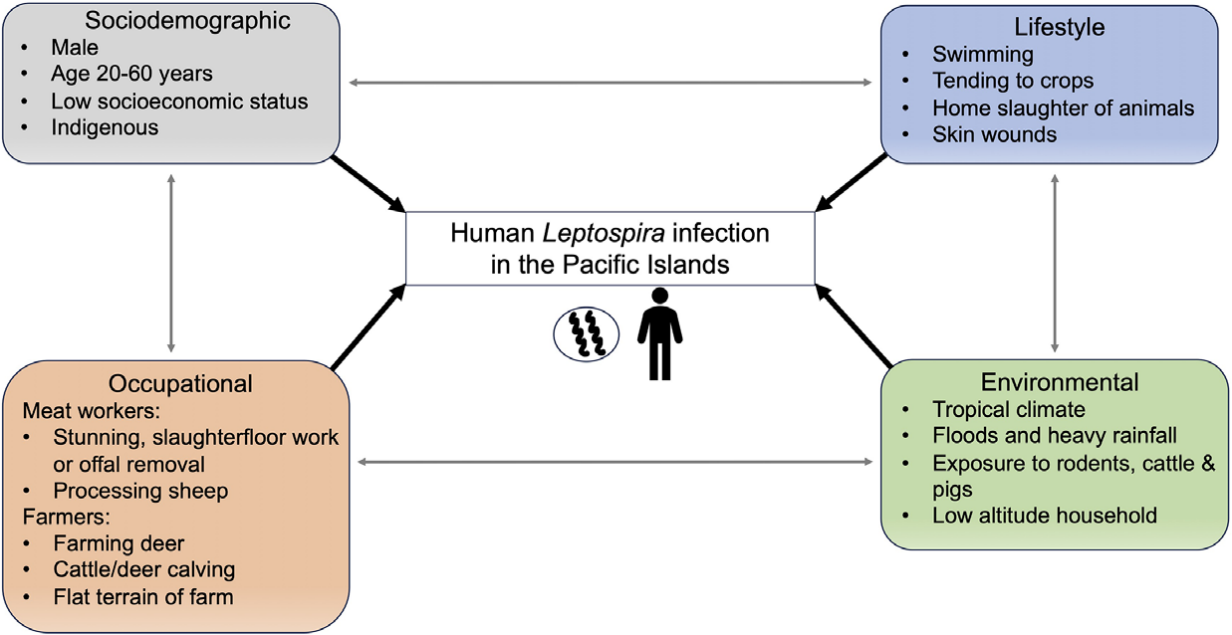
Primary drivers of human *Leptospira* infection in the Pacific Islands.

This review provides insight into the various drivers of human *Leptospira* infection in the Pacific Islands. The identified drivers of infection were predominantly occupational in the temperate climate of New Zealand, whereas in the tropical/sub-tropical Pacific Islands, exposures were attributed to a combination of factors including low socioeconomic status, agricultural activity, water-associated exposures, diverse mammalian reservoirs, and weather events. The main drivers of infection identified in this review are shown in Figure 3.

### Significant drivers of human Leptospira infection in the Pacific Islands

In terms of socio-demographics, males aged between 20 and 60 years were at the highest risk of *Leptospira* infection in the Pacific Islands, which is consistent with the global literature [1]. A higher infection risk was identified in Indigenous populations and may be linked to cultural differences in animal husbandry or slaughtering practices [38]. Poverty was another important driver of infection in remote Pacific Islands, which aligns with findings from other resource-limited settings in Asia and South America [54]. Low socioeconomic areas are associated with more intimate contact with animals and poor sanitation and hygiene [3]. Furthermore, untreated household water supply systems were independently associated with infections, which may be due to high rates of bacterial contamination in these water sources [55].

Similar to other temperate regions like Europe [56], *Leptospira* infection in New Zealand was primarily associated with occupational exposures [51]. High-risk abattoir roles were stunning, working on the slaughterfloor and offal removal. Stunning animals can cause splashing of urine, and both slaughterfloor workers and those in offal removal directly handle animal viscera [30]. Offal removal is also labour-intensive, resulting in skin wounds that may facilitate the transmission of bacteria [16]. Sheep abattoir workers were at the highest risk of infection in recent studies, with workers reporting that sheep tended to urinate spontaneously when stunned [29]. In older studies, pig workers had an increased infection risk; however, dry stock vaccinations were not available at the time [21]. Interestingly, PPE use did not reduce the risk of *Leptospira* infection in workers and one study reported an increased risk of infection with facemask and safety goggles’ use [29]. Whilst the finding of an increased infection risk with PPE use was obtained from unadjusted estimates and thus likely influenced by confounding factors [29], the lack of reduction in infection risk with PPE use across studies warrants further investigation. A possible reason may be poor compliance, as workers stated that they often lifted up their safety goggles or masks to remove sweat or fog to restore visibility [29]. These findings warrant further investigation into the use of PPE in abattoir workers. In farm workers, farming deer was associated with a higher risk of infection compared to farming other animals [46]. Deer are not used to being handled by farmers, and they were reported to dribble urine whilst being handled [46]. Additional factors associated with *Leptospira* infection in farm workers were assisting in cattle and deer calving, which would place workers into close contact with animal urine and placental tissue, and having a farm with flat terrain, which may increase the presence of standing puddles that accumulate leptospires [46]. For veterinarians, home slaughter of animals was a significant driver of infection [47], which could be linked to a lack of familiarity with appropriate hygiene practices for animal slaughter.

In tropical Pacific Islands, farmers were an at-risk occupational group [25], and accordingly, rural agricultural areas were geographical hotspots for infection [14, 25, 33, 38, 43]. Students were another high-risk group, which is likely attributed to their recreational exposures [26, 34]. Freshwater swimming was a significant lifestyle driver of infection across tropical Pacific Islands and has been recognized as a high-risk activity for leptospirosis in the literature due to the ability of leptospires to remain virulent in freshwater for several weeks [55]. Furthermore, gardening was associated with infections in the Federated States of Micronesia [26], which brings individuals into close contact with rodents and soil and water contaminated with their urine. In addition, open skin wounds may predispose individuals to infection, facilitating the transmission of leptospires to humans by compromising the physical dermal barrier [57].

The predominant animal reservoirs for infection in tropical Pacific Islands were rodents and livestock (especially cattle and pigs). Traditionally, livestock are recognized as important hosts of leptospirosis in rural areas and rodents are the main reservoirs in urban areas [54]. The remote Pacific Islands represent a unique situation with frequent exposure to both rodents and livestock due to the integration of agriculture into urban residences, for example, through backyard piggeries [36] and household crops [5].

In terms of seasonality, higher leptospirosis incidence occurred during the wet season in tropical Pacific Islands [20]. Similar findings have been reported in other tropical locations, including Far North Queensland in Australia [58] and Sri Lanka [59]. During the wet season, there are dissemination of leptospires from rainfall [60], an increase in recreational water-associated exposures, and the proliferation of rodent populations during their breeding season [61]. In the Pacific Islands, the highest risk of infection was in the one-to-two-month period following heavy rainfall events [24, 42]. Short periods of intense rainfall result in the accumulation of leptospires in soil and water at the surface level [55] whilst simultaneously contaminating water supply systems and displacing people from their homes and animals from their habitats, transiently increasing human–animal interactions [3].

The abundance of backyard piggeries in the remote Pacific islands additionally poses a distinct risk to these communities. Piggeries are typically built adjacent to rivers which allow drainage of waste but also result in contaminated water travelling downstream towards other households [38].

### Implications for mitigation strategies

To target the main drivers of infection identified in this review, mitigation strategies can be implemented at the regional, community, and individual levels.

At a regional level, improving the disease reporting would enhance our understanding of leptospirosis epidemiology. MAT and PCR diagnosis require the transport of specimens to laboratories with specialized equipment [62], which is often not feasible in remote Pacific Islands. Hence, the use of more readily available tests such as IgM ELISA and IgM rapid immunochromatography test (e.g. Leptocheck-WB) may help to improve disease reporting and response to outbreaks. These tests have demonstrated high diagnostic sensitivity during acute leptospirosis illness [62] and are now being used for initial diagnosis in French Polynesia [24], Wallis and Futuna [41], Vanuatu [44], and Fiji [52]. In addition, greater collaboration with veterinary surveillance systems for leptospirosis could help to inform human infection risk and public health response [63].

Furthermore, increasing the use of animal vaccinations against leptospirosis could further reduce disease prevalence in animal reservoirs, particularly in New Zealand. Whilst there are compulsory vaccination programmes for dairy cattle and pigs, there remains limited uptake of the available vaccines for sheep and deer [64]. Given the risk of infection associated with both sheep and deer handling, promoting the uptake of these vaccinations could further reduce transmission risk for New Zealand workers.

At a community level, improving water security, pest control, and piggery management could significantly reduce the leptospirosis burden in remote Pacific Islands. Climate-resilient water sanitation projects have been implemented with success in the Solomon Islands [65] and should be promoted in other islands to reduce the risk of household water supply contamination. Local communities can also contribute by maintaining sewage systems and preventing the accumulation of debris in waterways [3]. Improved waste management would assist in controlling rodent populations, and additional measures such as rat trapping, removing nesting places, and limiting rodent access to food could be implemented during rodent breeding months [61]. Furthermore, proper management of piggery waste and aiming to build piggeries further away and downhill from homes could mitigate the risk for households located nearby and below piggeries [35].

Further adjustments to occupational practices in New Zealand and targeted information campaigns in remote Pacific Islands could address the individual-level drivers of infection. In abattoirs, employers could introduce regular PPE compliance audits and institute additional measures such as fit testing for facemasks and safety goggles. To reduce the infection risk for slaughterboard workers, stunned animals could be placed on a separate platform [29], or on disposable covers, to minimize the accumulation of urine. For workers in offal removal, the use of cut-resistant gloves could decrease the risk of lacerations to workers’ hands [66]. In remote Pacific Islands, public health media campaigns could promote messages about simple hygiene risks (e.g. ‘wash wounds with clean water and soap, and cover up’ and ‘use boots and gloves when gardening or in the field’), reducing rodent infiltration (‘avoid leaving rubbish scattered around the home’) [63] and minimizing freshwater exposures (‘avoid swimming in puddles and rivers after heavy rain’). Furthermore, in New Caledonia and French Polynesia, leptospirosis education has been incorporated into the primary school curriculum through posters and board games [63], and other Pacific Islands could utilize similar approaches to promote preventative behaviours from a young age. In addition, collaboration with Indigenous community leaders is required to identify and address cultural activities that may contribute to a higher infection risk.

### Limitations

This review provides important insight into the drivers of human *Leptospira* infection in the Pacific Islands; however, there were some limitations present. Given the broad nature of this review, there was significant heterogeneity of included studies, and hence, meta-analysis was not conducted, and instead, study findings were synthesized descriptively. Furthermore, the MEVORECH tool is validated for risk factor studies but was originally designed for chronic diseases [12], and hence, potential biases identified in domains such as the reference period for outcomes and/or exposures may be attributed to the limited applicability of the tool to acute infectious disease studies. Lastly, several studies in this review included probable cases diagnosed using indirect methods, such as ELISA and MAT, often with differing antibody titre cut-off levels, and sometimes only reported unadjusted estimates, which made it challenging at times to identify the true drivers of infection.

### Directions for future research

Future research on human leptospirosis in the Pacific Islands designed to minimize bias is essential to further our knowledge of local disease epidemiology. There is a need for greater focus on serogroup-specific drivers of *Leptospira* infection and studies at a regional level that compare findings in each Pacific Island to individualize mitigation strategies. Furthermore, research into PPE use in meat workers is required given the limited effectiveness identified in this review.

It is also important to consider how climate change may exacerbate existing drivers of leptospirosis in the region. The Pacific represents one of the most natural disaster-prone regions in the world [65], and the predicted increase in floods and cyclones may increase opportunities for *Leptospira* infection by disrupting local ecosystems, contaminating water sanitation networks, and damaging human infrastructure [3]. We advocate for ecosystem-based adaptation approaches, such as mangrove conservation and restoration projects, which can provide coastal flood protection and useful economic and environmental co-benefits for Pacific Island communities [67, 68]. With a significant number of leptospirosis outbreaks after flooding being reported worldwide, the mitigation strategies developed by the Pacific islands may help to inform the actions of other affected regions.

## Conclusions

The drivers of human *Leptospira* infection in the Pacific Islands are multifactorial and differ significantly between New Zealand and the tropical Pacific Islands. The drivers of infection in New Zealand exemplify the predominance of occupational risks in temperate climates, and public health interventions should accordingly target abattoir and farm workers. In tropical Pacific Islands, infections are the product of distinct environmental characteristics, diverse animal reservoirs, and human activity that facilitate leptospirosis transmission. The drivers of infection identified in this review are not necessarily distinct from factors identified in other parts of the world; however, it is the complexity and multifaceted nature of leptospirosis transmission that is unique to the region, particularly in the face of climate change. Leptospirosis is ultimately a preventable disease, but further research is required to untangle the ecological mechanisms that underlie disease transmission in the Pacific Islands.

## Supporting information

Kharwadkar et al. supplementary material Kharwadkar et al. supplementary material

## Data Availability

All relevant data are provided in text and in the Supplementary Material.

## Supplementary material

For supplementary material accompanying this paper visit http://doi.org/10.1017/S0950268824001250. click here to view supplementary material
