# Supplementary material for: Drivers of human Leptospira infection in the Pacific Islands: A systematic review
Source: Epidemiol Infect. 2024 Oct 8;152:e118. doi: 10.1017/S0950268824001250 (PMC11474764; doi:10.1017/S0950268824001250)
Supplement: Kharwadkar et al. supplementary material [file S0950268824001250sup001.docx]

**Supplementary Material**

Contents:

**S1:** Search strategy

**Table S1:** Adapted Methodological Evaluation of Observational Research Checklist (MEVORECH) tool for risk of bias assessment

**Table S2:** Findings related to sociodemographic drivers of human *Leptospira* infection

**Table S3:** Findings related to occupational drivers of human *Leptospira* infection

**Table S4:** Findings related to lifestyle drivers of human *Leptospira* infection

**Table S5:** Findings related to environmental drivers of human *Leptospira* infection

**S1:** Search strategy

Ovid MEDLINE:

(Exp Leptospirosis/ OR (Leptospir* OR Weil* disease OR Stuttgart disease OR Mud fever OR Rice-field fever OR Rice field fever OR Cane-cutter disease OR Cane cutter disease OR Swineherd* disease OR Pea picker disease* OR Canicola fever).ti,ab,kw.) AND (Exp Pacific islands/ OR (Pacific OR Melanesia OR Fiji OR New Caledonia OR Papua New Guinea OR Solomon Island* OR Vanuatu OR New Hebrides OR Micronesia OR Guam OR Kiribati OR Gilbert Island* OR Banaba OR Christmas Island OR Kiritimati OR Marshall Island* OR Enewetak OR Bikini OR Rongelap OR Kwajalein OR Majuro OR Federated States of Micronesia OR Yap OR Chuuk OR Pohnpei OR Kosrae OR Nauru OR Northern Mariana Island* OR Saipan OR Palau OR Belau OR Baker Island OR Howland Island OR Jarvis Island OR Johnston Atoll OR Kingman Reef OR Midway Atoll OR Palmyra Atoll OR Wake Island OR Polynesia OR Samoa OR New Zealand OR Aotearoa OR Cook island* OR Easter Island OR Rapa Nui OR French Polynesia OR Tahiti OR Society Island* OR Marquesas Island* OR Austral Island* OR Tuomotu OR Gambier Island* OR Tuamotu Island* OR Hawaii OR Niue OR Pitcairn OR Tokelau OR Tonga OR Tuvalu OR Wallis OR Futuna).ti,ab,kw.)

Scopus:

#1: TITLE-ABS-KEY ( ( leptospir* OR "Weil* disease" OR "Stuttgart disease" OR "Mud fever" OR "Rice-field fever" OR "Rice field fever" OR "Cane-cutter disease" OR "Cane cutter disease" OR "Swineherd* disease" OR "Pea picker* disease" OR "Canicola fever" ) AND ( pacific OR melanesia OR fiji OR "New Caledonia" OR "Papua New Guinea" OR "Solomon Island*" OR vanuatu OR "New Hebrides" OR micronesia OR guam OR kiribati OR "Gilbert Island*" OR banaba OR "Christmas Island" OR kiritimati OR "Marshall Island*" OR enewetak OR bikini OR rongelap OR kwajalein OR majuro OR "Federated States of Micronesia" OR yap OR chuuk OR pohnpei OR kosrae OR nauru OR "Northern Mariana Island*" OR saipan OR palau OR belau OR "Baker Island" OR "Howland Island" OR "Jarvis Island" OR "Johnston Atoll" OR "Kingman Reef" OR "Midway Atoll" OR "Palmyra Atoll" OR "Wake Island" OR polynesia OR samoa OR "New Zealand" OR aotearoa OR "Cook Island*" OR "Easter Island" OR "Rapa Nui" OR "French Polynesia" OR tahiti OR "Society Island*" OR "Marquesas Island*" OR "Austral Island*" OR tuomotu OR "Gambier Island*" OR "Tuamotu Island*" OR hawaii OR niue OR pitcairn OR tokelau OR tonga OR tuvalu OR wallis OR futuna ) )

#2: TITLE-ABS-KEY ( Leptospir* OR "Weil* disease" OR "Stuttgart disease" OR "Mud fever" OR "Rice-field fever" OR "Rice field fever" OR "Cane-cutter disease" OR "Cane cutter disease" OR "Swineherd* disease" OR "Pea picker* disease" OR "Canicola fever" ) AND ( LIMIT-TO ( AFFILCOUNTRY , "New Zealand" ) OR LIMIT-TO ( AFFILCOUNTRY , "New Caledonia" ) OR LIMIT-TO ( AFFILCOUNTRY , "Fiji" ) OR LIMIT-TO ( AFFILCOUNTRY , "French Polynesia" ) OR LIMIT-TO ( AFFILCOUNTRY , "Wallis and Futuna" ) OR LIMIT-TO ( AFFILCOUNTRY , "Federated States of Micronesia" ) OR LIMIT-TO ( AFFILCOUNTRY , "Vanuatu" ) OR LIMIT-TO ( AFFILCOUNTRY , "American Samoa" ) OR LIMIT-TO ( AFFILCOUNTRY , "Guam" ) OR LIMIT-TO ( AFFILCOUNTRY , "Papua New Guinea" ) )

Note: Searches #1 and #2 were performed separately as they were not able to be combined appropriately using the Boolean operator “OR”.

Web of Science Core Collection:

#1: TS=((Leptospir* OR "Weil* disease" OR "Stuttgart disease" OR "Mud fever" OR "Rice-field fever" OR "Rice field fever" OR "Cane-cutter disease" OR "Cane cutter disease" OR "Swineherd* disease" OR "Pea picker* disease" OR "Canicola fever" ) AND ( Pacific OR Melanesia OR Fiji OR "New Caledonia" OR "Papua New Guinea" OR "Solomon Island*" OR Vanuatu OR "New Hebrides" OR Micronesia OR Guam OR Kiribati OR "Gilbert Island*" OR Banaba OR "Christmas Island" OR Kiritimati OR "Marshall Island*" OR Enewetak OR Bikini OR Rongelap OR Kwajalein OR Majuro OR "Federated States of Micronesia" OR Yap OR Chuuk OR Pohnpei OR Kosrae OR Nauru OR "Northern Mariana Island*" OR Saipan OR Palau OR Belau OR "Baker Island" OR "Howland Island" OR "Jarvis Island" OR "Johnston Atoll" OR "Kingman Reef" OR "Midway Atoll" OR "Palmyra Atoll" OR "Wake Island" OR Polynesia OR Samoa OR "New Zealand" OR Aotearoa OR "Cook Island*" OR "Easter Island" OR "Rapa Nui" OR "French Polynesia" OR Tahiti OR "Society Island*" OR "Marquesas Island*" OR "Austral Island*" OR Tuomotu OR "Gambier Island*" OR "Tuamotu Island*" OR Hawaii OR Niue OR Pitcairn OR Tokelau OR Tonga OR Tuvalu OR Wallis OR Futuna ))

#2: TS=(Leptospir* OR “Weil* disease” OR “Stuttgart disease” OR “Mud fever” OR “Rice-field fever” OR “Rice field fever” OR “Cane-cutter disease” OR “Cane cutter disease” OR “Swineherd* disease” OR “Pea picker* disease” OR “Canicola fever”)

#3: Refine #2 by Countries/Regions: NEW ZEALAND or NEW CALEDONIA or FIJI or MICRONESIA or PAPUA N GUINEA or VANUATU or SAMOA

#1 OR #3

Ovid Embase:

(Exp Leptospirosis/ OR (Leptospir* OR Weil* disease OR Stuttgart disease OR Mud fever OR Rice-field fever OR Rice field fever OR Cane-cutter disease OR Cane cutter disease OR Swineherd* disease OR Pea picker disease* OR Canicola fever).ti,ab,kw.) AND (Exp Pacific islands/ OR (Pacific OR Melanesia OR Fiji OR New Caledonia OR Papua New Guinea OR Solomon Island* OR Vanuatu OR New Hebrides OR Micronesia OR Guam OR Kiribati OR Gilbert Island* OR Banaba OR Christmas Island OR Kiritimati OR Marshall Island* OR Enewetak OR Bikini OR Rongelap OR Kwajalein OR Majuro OR Federated States of Micronesia OR Yap OR Chuuk OR Pohnpei OR Kosrae OR Nauru OR Northern Mariana Island* OR Saipan OR Palau OR Belau OR Baker Island OR Howland Island OR Jarvis Island OR Johnston Atoll OR Kingman Reef OR Midway Atoll OR Palmyra Atoll OR Wake Island OR Polynesia OR Samoa OR New Zealand OR Aotearoa OR Cook island* OR Easter Island OR Rapa Nui OR French Polynesia OR Tahiti OR Society Island* OR Marquesas Island* OR Austral Island* OR Tuomotu OR Gambier Island* OR Tuamotu Island* OR Hawaii OR Niue OR Pitcairn OR Tokelau OR Tonga OR Tuvalu OR Wallis OR Futuna).ti,ab,kw.)

**Table S1: Potential biases for adapted Methodological Evaluation of Observational Research Checklist (MEVORECH) tool [12] used for risk of bias assessment**

| **Adjusted domain of MEVORECH tool** | **Potential biases** |
| --- | --- |
| Source to measure human *Leptospira* infection | Not reported  Proxy reported (collected for the study)  Obtained from medical records (mining of data collected for health care purposes)  Obtained from administrative database (mining of data collected for health care purposes) |
| Reference period of human *Leptospira* infection | Reference period may be relevant but not included in definition of the outcome  Reference period different from recommended and not justified |
| Validation of methods to measure human *Leptospira* infection | Not reported  The authors reported inter-methods validation (one method vs. another)  The authors did not validate the methods to measure dependent variables (non-valid methods were obtained) |
| Reliability of methods to measure human *Leptospira* infection | Not reported  Intra-observer variability is reported with subjective judgment of reliability  Inter-observer variability is reported with subjective judgment of reliability |
| Masking of exposure status for investigators who measured human *Leptospira* infection | Not reported  Was possible but not obtained |
| Source to measure the driver(s) of infection | Not reported  Proxy reported (collected for the study)  Obtained from medical records (mining of data collected for health care purposes)  Obtained from administrative database (mining of data collected for health care or environmental purposes) |
| Reference period/length of exposure to the driver(s) of infection | Reference period/length of exposure may be relevant but not included in definition of the exposure  Reference period/length of exposure different from recommended and not justified |
| Intensity/dose of exposure to the driver(s) of infection | Intensity/dose can be relevant but not assessed in the study |
| Validation of estimates for the driver(s) of infection* | Not reported  The authors reported inter-methods validation (one method vs. another)  The authors did not validate the methods to measure exposure (risk factors, independent variables) |
| Reliability of estimates for the driver(s) of infection* | Not reported  Intra-observer variability is reported with subjective judgment of reliability |
| Assessment of confounding factors or factors that can modify the association between the driver(s) and human *Leptospira* infection | Not reported  Major confounding factors/effect modifiers were not assessed  Major confounding factors /effect modifiers were assessed partially |
| Validity of methods to measure confounding factors | Not reported  Unknown validity to measure confounding factors  Non valid methods to measure confounding factors |
| Statistical methods to reduce bias | Not reported  The authors did not obtain methods to reduce bias |
| Appropriateness of statistical model to reduce bias | Strategies to reduce research specific bias not reported  Authors did not use statistical models that may be the most appropriate according to the published literature  Authors did not justify choice of statistical models to reduce research specific bias  Authors attempted to reduce bias in post hoc statistical adjustment |
| Reporting of tested hypothesis | Unclear reporting of the estimates  Crude estimates without adjustment for confidential sources of bias  Incomplete selective reporting of the tested hypotheses (compared to aim and objectives) |
| Precision of the estimates | Numeric value of estimates not reported (p value only, significance or non-significance only)  Mean only reported without p value or variance |
| Sample size justification | Not reported  Justification by authors is incomplete or inaccurate  Post-hoc analyses |

Note: if studies did not demonstrate any of the potential biases listed for a domain, they were considered to have a low risk of bias for the respective domain.

*Authors’ estimates for age, gender/sex, ethnicity and seasonality were considered to be valid and reliable

**Table S2:** Findings related to sociodemographic drivers of human *Leptospira* infection

| **Citation details** | **Study location** | **Reported Study period** | **Study population** | **Sample size** | **Leptospirosis outcome(s)** | **Covariates in model** | **Findings** |
| --- | --- | --- | --- | --- | --- | --- | --- |
| **Age** |  |  |  |  |  |  |  |
| [19] | New Zealand | February- March 2008 | Slaughterhouse workers | 242 sampled  23 seropositive | Seroprevalence | None | **Seropositive workers in 41-53 years old age tercile v 18-40 years old age tercile OR 4.79 (95% CI: 1.15-32.48) (p-value NR)**  **Seropositive workers in 54-69 year old age tercile v 18-40 year old age tercile OR 6.94 (95% CI: 1.84-45.37) (p-value NR)** |
| [20] | Palau, Guam, Federated States of Micronesia, Vanuatu, Fiji, Tonga, Wallis, Futuna and French Polynesia | September 2003-December 2005 | Hospital patients | 263 suspected  69 confirmed cases | Incidence (confirmed cases only) | None | Ages of confirmed patients v unconfirmed patients NS (p-value NR)  Futuna: infected patients were significantly younger (p=0.05, authors reported as significant) |
| [25] | New Caledonia | January 1985-December 1986 | General population | Total population 145368  193 cases | Incidence; seroprevalence | None | **<20 year olds less affected v other ages χ^2^(1)=78.95 (p<0.05)**  **20-59 year olds more affected v 20-29 year olds χ^2^(1)=7.30 (p<0.05)**  **20-59 year olds more affected v 30-39 year olds χ^2^(1)=8.22 (p<0.05)**  **20-59 year olds more affected v 40-49 year olds χ^2^(1)=60.29 (p<0.05)**  **20-59 year olds more affected v 50-59 year olds χ^2^(1)=13.61 (p<0.05)**  **40-49 years olds more affected v 20-29 year olds χ^2^(1)=12.16 (p<0.05)**  **40-49 years olds more affected v 30-39 year olds χ^2^(1)=9.87 (p<0.05)**  40-49 years olds more affected v 50-59 year olds χ^2^(1)= 2.39 (p>0.05) |
| [14] (Study 1) | New Caledonia (Nera) | January 1985-December 1986 | General population | Total population 3410  60 cases | Incidence | None | **<20 year olds least affected v other ages χ^2^(1)=16.20 (p<0.001)** |
| [14] (Study 3) | New Caledonia (Coulée) | January 1985-December 1986 | General population | Total population 14614 26 cases | Incidence | None | **<20 year olds least affected v other ages χ^2^(1)=12.82 (p<0.001)** |
| [26] | Federated States of Micronesia | June-September 2011 | Hospital patients | 54 tested  11 confirmed cases | Incidence (confirmed cases only) | None | Cases in 10-24 years old age group v all other ages OR 4.0 (95% CI 1.0-16.2, p=0.077) |
| [15] | New Zealand | February 2008-May 2011 | Abattoir workers | 592 participants  51 new infections | Incidence | None | New infection in 40-50 year olds v <40 year olds RR 0.9 (95% CI 0.4-2.3, p=0.871)  New infection in 50-57.5 year olds v <40 year olds RR 1.6 (95% CI 0.7-3.6, p=0.252)  New infection in >57.5 year olds v <40 year olds RR 1.3 (95% CI 0.6-3.1, p=0.486) |
| [29] | New Zealand | November 2009-March 2010 | Beef abattoir workers | 567 participants  62 seropositive | Seroprevalence | None | **Crude odds of being seropositive increased linearly with age in beef workers (p<0.05)** |
| [29] | New Zealand | November 2009-March 2010 | Beef abattoir workers | 567 participants  62 seropositive | Seroprevalence | Work position | **Increasing age significant as a continuous variable in beef workers OR 1.1 (95% CI: 1.0-1.2, p=0.02)** |
| [30] | New Zealand | NR | Abattoir workers | 384 sampled  49 seropositive | Incidence | None | Reported same findings as [15] |
| [33] | Hawaii | January 1974-December 1998 | General population | Total population NR 709 cases (353 confirmed, 180 probable, 176 suspected) | Incidence (confirmed only) | None | **Cases in 0-9 year olds v 30-39 year olds RR 0.07 (95% CI: 0.03-0.16, p<0.0001)**  Cases in 10-19 year olds v 30-39 year olds RR 0.77 (95% CI: 0.54-1.10, p=0.18)  Cases in 20-29 year olds v 30-39 year olds RR 1.08 (95% CI: 0.80-1.45, p=0.18)  Cases in 40-49 year olds v 30-39 year olds RR 1.17 (95% CI: 0.85, 1.60, p=0.38)  Cases in 50-59 year olds v 30-39 year olds RR 0.65 (95% CI: 0.41-1.00, p=0.06)  **Cases in 60-69 year olds v 30-39 year olds RR 0.37 (95% CI: 0.21-0.62, p=0.00012)**  Cases in 70-79 year olds v 30-39 year olds RR 0.41 (0.20, 0.77, p=0.0054) |
| [33] | Hawaii | January 1974-December 1998 | General population | Total population NR 709 cases (353 confirmed, 180 probable, 176 suspected) | Incidence (confirmed only) | Island of exposure, ethnicity | **Cases in 0-9 year olds v 30-39 year olds** **OR 0.11 (95% CI: 0.03-0.29, p<0.0001)**  Cases in 10-19 year olds v 30-39 year olds OR 0.66 (95% CI: 0.35-1.19, p=0.17)  Cases in 50-59 year olds v 30-39 year olds OR 0.85 (95% CI: 0.42-1.58, p=0.6)  **Cases in 60-69 year olds v 30-39 year olds OR 0.46 (95% CI: 0.18-0.98, p=0.04)**  Cases in 70-79 year olds v 30-39 year olds OR 0.4 (95% CI: 0.11-1.03, p=0.06) |
| [34] | New Caledonia | January 2006-December 2016 | Notification data | Total population 268767 904 cases (700 confirmed, 204 probable) | Incidence (confirmed or probable) | None | **Infection with serogroup Pyrogenes in school-aged participants v older participants OR 2.126 (95% CI: 1.3054-3.4167, p=0.0016)** |
| [39] | Hawaii | April-March 2002 | Army blood bank donors | 488 participants  7 seropositive | Seroprevalence | None | **Seroprevalence in 18-30 years old age group v other ages (p<0.045)** |
| [41] | Wallis and Futuna | January 2008-June 2015 | Hospital patients | 338 suspected, 165 confirmed and 173 excluded cases | Incidence (suspected or confirmed) | None | **Seroconversion in males 10-30 years old v other ages RR 2.46 (p<0.001)**  **Seroconversion in females 40-59 years old v other ages RR 1.70 (p<0.001)** |
| [16] | New Zealand | February 2008-May 2011 | Sheep abattoir workers | 567 participants, 384 after follow-up (cases NR) | Incidence | None | Association between age (continuous) variable and cases OR 1.0 (95% CI: 0.9-1.0) (p-value NR) |
| [46] | New Zealand | May-October 2013 | Beef, sheep and deer farmers | 178 participants  12 seropositive | Seroprevalence | None | Association between age (continuous) variable and seroprevalence NS (p-value NR) |
| [48] | Hawaii | July 1988-June 1989 (Big Island); July-December 1988 (Kauai) | Hospital patients | Big Island: 172 participants, 123 followed, 20 seropositive  Kauai: 100 participants, 59 followed, 13 cases  Inferential statistics: 33 cases, 77 controls | Incidence | None | No difference between ages of cases and controls (p<0.58) |
| **Gender/Sex** |  |  |  |  |  |  |  |
| [19] | New Zealand | February- March 2008 | Slaughterhouse workers | 242 sampled  23 seropositive | Seroprevalence | None | **Seroprevalence in men v women OR 3.51 (95% CI: 1.27-12.40)** **(p-value NR)** |
| [20] | Palau, Guam, Federated States of Micronesia, Vanuatu, Fiji, Tonga, Wallis, Futuna and French Polynesia | September 2003-December 2005 | Hospital patients | 263 suspected  69 confirmed cases | Incidence (confirmed cases only) | None | **Cases more frequent in males v females at a regional level (p<0.05)**  Difference in cases between males v females NS in Futuna and the Marquesas (p-value NR) |
| [23] | New Zealand | NR | Dairy farm workers | 308 sampled  137 seropositive | Seroprevalence | None | **Hardjo: titres in males v females (p<0.001)**  **Pomona: titres in males v females (p<0.01)**  **Both serovars: titres in males v females (p<0.001)** |
| [24] | French Polynesia | January 2007-December 2017 | General population | Total population 280000 1365 cases (851 confirmed, 505 probable) | Incidence (annual incidence rates using confirmed and probable cases) | None | **Higher annual incidence rate in men v women RR 3.1 (95% CI: 2.7-3.5, p<0.001)** |
| [25] | New Caledonia | January 1985-December 1986 | General population | Total population 145368  193 cases | Incidence; seroprevalence | None | **Males more affected v females χ^2^(1)= 30.55 (p<0.05)** |
| [14] (Study 1) | New Caledonia (Nera) | January 1985-December 1986 | General population | Total population 3410  60 cases | Incidence | None | **Males more affected v females χ^2^(1)=15.02 (p<0.001)** |
| [14] (Study 3) | New Caledonia (Coulée) | January 1985-December 1986 | General population | Total population 14614 26 cases | Incidence | None | **Males more affected v females χ^2^(1)=9.116 (p<0.01)** |
| [26] | Federated States of Micronesia | June-September 2011 | Hospital patients | 54 tested  11 confirmed cases | Incidence (confirmed cases only) | None | Cases in males v females OR 3.6 (95%: CI 0.7-18.5, p=0.170) |
| [15] | New Zealand | February 2008-May 2011 | Abattoir workers | 592 participants  51 new infections | Incidence | None | Seroprevalence in males v females RR 1.9 (95% CI: 0.9-3.7, p=0.084) |
| [29] | New Zealand | November 2009-March 2010 | Sheep abattoir workers | 567 participants  62 seropositive | Seroprevalence | None | **Increased odds of seropositivity in males v females OR 6.4 (95% CI: 1.9-21.1, p=0.003)** |
| [29] | New Zealand | November 2009-March 2010 | Sheep abattoir workers | 567 participants  62 seropositive | Seroprevalence | Work position, years worked at meat plant, meat plant | Increased odds of seropositivity males v females OR 3.1 (95% CI: 0.8-11.7, p=0.089) |
| [30] | New Zealand | NR | Abattoir workers | 384 sampled  49 seropositive | Incidence | None | Reported same findings as [15] |
| [31] | New Zealand | 2010-2015 | General population | Total population NR  442 cases | Incidence | None | **Significant differences in cases between males v females, where 90% of cases were males (p<0.001)** |
| [35] | American Samoa | May-July 2010 | General population | 807 participants (number seropositive NR) | Seroprevalence | May include occupational group, house below median altitude of village, vegetation type, soil type, piggeries within 250 metres and above house | **Seroprevalence in** **males v females OR 2.77 (95% CI: 1.74-4.42) (p-value NR)** |
| [36] | American Samoa | May-July 2010 | General population | 807 participants  125 seropositive | Seroprevalence | None | **Seroprevalence in males v females OR 3.06 (95% CI: 2.00-4.71) (p-value NR)** |
| [36] | American Samoa | May-July 2010 | General population | 807 participants  125 seropositive | Seroprevalence | Heard of leptospirosis, occupation, piggeries within 250 metres and above house. | **Seroprevalence in males v females OR 3.40 (95% CI: 2.18-5.33) (p-value NR)** |
| [38] | Fiji | September-December 2013 | General population | 2152 participants  417 seropositive | Seroprevalence | None | **Seroprevalence in males v females OR 1.67 (95% CI: 1.35-2.08) (p-value NR)** |
| [38] | Fiji | September-December 2013 | General population | 2152 participants  417 seropositive | Seroprevalence | Ethnicity, community type, metered water available at home, work location | **Seroprevalence in males v females OR 1.55 (95% CI: 1.16-2.08, p=0.03)** |
| [39] | Hawaii | April-March 2002 | Army blood bank donors | 488 participants  7 seropositive | Seroprevalence | None | **Female gender was more common in seropositive v seronegative groups (p<0.034)** |
| [40] | New Zealand | Not reported | Dairy farm workers | 226 participants, 213 sampled  84 seropositive | Seroprevalence | None | **Hardjo: seroprevalence in males v females (p<0.01)**  **Both serovars: seroprevalence in male v females (p<0.01)** |
| [44] | Vanuatu | January 2013-August 2014 | Hospital patients | 161 patients  12 cases (5 confirmed, 7 probable)  29 seropositive | Seroprevalence | None | Seroprevalence in males v females OR 1.7 (95% CI: 0.75-3.84, p=0.2) |
| [44] | Vanuatu | January 2013-August 2014 | Hospital patients | 161 patients  12 cases (5 confirmed, 7 probable)  29 seropositive | Seroprevalence | Water at home, freshwater fishing, contact with surface water, hunting, contact with animals (pigs, dogs/cats, horses/goats/sheep, cattle), rodents seen in vicinity, island of residence, occupational risk | Seroprevalence in males v females OR 0.69 (95% CI: 0.21-2.26, p=0.54) |
| [45] | New Caledonia | 1989 | General population | Total population 165000  144 cases | Incidence | None | **Incidence in men v women incidence rate ratio 2.29 (p<0.001)** |
| [16] | New Zealand | February 2008-May 2011 | Sheep abattoir workers | 567 participants, 384 after follow-up (cases NR) | Incidence | None | New infection in males v females OR 1.3 (95% CI: 0.6-3.0, p=0.459) |
| [16] | New Zealand | February 2008-May 2011 | Sheep abattoir workers | 567 participants, 384 after follow-up (cases NR) | Incidence | Work position, abattoir plant | New infection in males v females OR 0.5 (95% CI: 0.2-1.4) (p-value NR) |
| [47] | New Zealand | May/June 2012 | Veterinarians | 277 participants  14 seropositive | Seroprevalence | None | Seroprevalence in men v women (p=0.40) |
| [48] | Hawaii | July 1988-June 1989 (Big Island); July-December 1988 (Kauai) | Hospital patients | Big Island: 172 participants, 123 followed, 20 seropositive  Kauai: 100 participants, 59 followed, 13 cases  Inferential statistics: 33 cases, 77 controls | Incidence | None | NS between cases and controls based on sex (p<0.93) |
| [49] | New Zealand | Not reported | Pig farmers | 70 participants  20 seropositive | Seroprevalence | None | Difference in seroprevalence based on gender reported NS (p-value NR) |
| [51] | New Zealand | 1990-1998 | General population | Total population NR  1397 cases | Incidence | None | **Livestock workers: incidence in males v females RR 6.6 (95% CI: 4.6-9.4) (p-value NR)**  Meat processing workers: incidence in males v females RR 1.1 (95% CI: 0.8-1.6) (p-value NR)  Forestry workers: incidence in males v females RR 1.2 (95% CI: 0.2-8.7) (p-value NR) |
| **Ethnicity** |  |  |  |  |  |  |  |
| [22] | New Zealand | 1979-1980 | Meat inspectors and workers | Inspectors: 1215 sampled, 121 seropositive  Workers: 1248 sampled, 77 seropositive | Seroprevalence | None | **Seroprevalence in Pacific Islanders v other races (p<0.0001)** |
| [25] | New Caledonia | January 1985-December 1986 | General population | Total population 145368  193 cases | Incidence; seroprevalence | None | Incidence in Europeans v Melanesians χ^2^(1)=1.80 (p>0.05)  Incidence in Melanesian v Wallisians χ^2^ (1)=1.06 (p>0.05) |
| [14] (Study 1) | New Caledonia (Nera) | January 1985-December 1986 | General population | Total population 3410  60 cases | Incidence | None | Incidence in Europeans v Melanesians χ^2^(1)=1.37 (p=0.3) |
| [14] (Study 3) | New Caledonia (Coulée) | January 1985-December 1986 | General population | Total population 14614 26 cases | Incidence | None | Incidence in Europeans v Melanesians χ^2^(1)=1.98 (p>0.1) |
| [15] | New Zealand | February 2008-May 2011 | Abattoir workers | 592 participants  51 new infections | Incidence | None | New infection in Māori workers v New Zealand-Europeans RR 1.6 (95% CI:0.9-3.0, p=0.132)  New infection in other workers v New Zealand-Europeans RR 1.4 (95% CI: 0.5-4.3, p=0.541) |
| [30] | New Zealand | NR | Abattoir workers | 384 sampled  49 seropositive | Incidence | None | Reported same findings as [15] |
| [33] | Hawaii | January 1974-December 1998 | General population | Total population NR 709 cases (353 confirmed, 180 probable, 176 suspected) | Incidence (confirmed only) | None | Cases in Hawaiians v Caucasians RR 0.81 (95% CI: 0.55-1.16, p=0.29)  **Cases in Japanese v Caucasians RR 0.37 (95% CI: 0.24-0.54, p<0.0001)**  **Cases in Filipino v Caucasians RR 0.22 (95%CI: 0.11-0.38, p<0.0001)**  Cases in Samoan v Caucasians RR 1.01 (95% CI: 0.36-2.29, p=1.00)  **Cases in Chinese v Caucasians Rr 0.09 (95% CI: 0.01-0.30, p<0.0001)** |
| [33] | Hawaii | January 1974-December 1998 | General population | Total population NR 709 cases (353 confirmed, 180 probable, 176 suspected) | Incidence (confirmed only) | Age group, island of exposure | Cases in Hawaiians v Caucasians OR 0.78 (95% CI:0.50-1.18, p=0.25)  **Cases in Japanese v Caucasians OR 0.50 (95% CI: 0.31-0.78, p=0.002)**  **Cases in Filipino v Caucasians OR 0.23 (95% CI: 0.11-0.44, p<0.0001)** |
| [38] | Fiji | September-December 2013 | General population | 2152 participants  417 seropositive | Seroprevalence | None | **Seroprevalence in iTaukei v Indo-Fijians OR 1.66 (95% CI:2.53-5.29)** **(p-value NR)**  **Seroprevalence in other ethnicities v Indo-Fijian OR 3.23 (95% CI: 1.38-7.56) (p-value NR)** |
| [38] | Fiji | September-December 2013 | General population | 2152 participants  417 seropositive | Seroprevalence | Gender, community type (i.e., urban v rural), metered water available at home, work location | **Seroprevalence in** **iTaukei v Indo-Fijians OR 3.51 (95% CI: 2.23-5.54, p<0.001)**  Seroprevalence in other ethnicities v Indo-Fijians OR 2.32 (95% CI: 0.82-6.58, p=0.114) |
| **Household water supply** |  |  |  |  |  |  |  |
| [38] | Fiji | September-December 2013 | General population | 2152 participants  417 seropositive | Seroprevalence | None | **Seroprevalence in households without metered water at home v households with metered water at home OR 1.92 (95% CI: 1.54-2.39) (p-value NR)** |
| [38] | Fiji | September-December 2013 | General population | 2152 participants  417 seropositive | Seroprevalence | Gender, ethnicity, community type (i.e., urban v rural), work location | **Households without metered water at home v households with metered water at home OR 1.52 (95% CI:1.14-2.03, p=0.004)** |
| [44] | Vanuatu | January 2013-August 2014 | Hospital patients | 161 patients  12 cases (5 confirmed, 7 probable)  29 seropositive | Incidence (confirmed or probable cases), Seroprevalence | None | **Seroprevalence in participants with water from well or natural source at home v not from well or natural source OR 4.19 (95% CI: 1.5-11.69, p=0.006)** |
| [44] | Vanuatu | January 2013-August 2014 | Hospital patients | 161 patients  12 cases (5 confirmed, 7 probable)  29 seropositive | Incidence (confirmed or probable cases), Seroprevalence | Sex, freshwater fishing, contact with surface water, hunting, contact with animals (pigs, dogs/ cats, horses /goats/ sheep, cattle), rodents seen in vicinity, island of residence, occupational risk | Seroprevalence in participants with water from well or natural source at home v not from well or natural source OR 2.05 (95% CI: 0.46-9.17, p=0.34) |
| [48] | Hawaii | July 1988-June 1989 (Big Island); July-December 1988 (Kauai) | Hospital patients | Big Island: 172 participants, 123 followed, 20 seropositive  Kauai: 100 participants, 59 followed, 13 cases  Inferential statistics: 33 cases, 77 controls | Incidence | None | **Higher percentage of cases in participants with household water catchment system v no household water catchment system (p=0.003)** |
| **Poverty** |  |  |  |  |  |  |  |
| [36] | American Samoa | May-July 2010 | General population | 807 participants  125 seropositive | Seroprevalence | None | **Seroprevalence in participants with annual household income 20-30,000 USD v >30,000 USD OR 3.20 (95% CI: 1.06-9.60) (p-value NR)**  Seroprevalence in participants with annual household income 10-20,000 USD v >30,000 USD OR 2.05 (95% CI: 0.77-5.47) (p-value NR)  **Seroprevalence in participants with annual household income <10,000 USD v >30,000 USD OR 2.74 (95% CI: 1.05-7.11)** **(p-value NR)**  Seroprevalence in participants with annual household income not declared v >30,000 USD OR 1.22 (95% CI: 0.41-3.63) (p-value NR) |
| [36] | American Samoa | May-July 2010 | General population | 807 participants  125 seropositive | Seroprevalence | Male, heard of leptospirosis, occupation, piggeries within 250 metres and above house | **Seroprevalence in participants with annual household income 20-30,000 USD v >30,000 USD OR 3.67 (95% CI: 1.18-11.40) (p-value NR)**  Seroprevalence in participants with annual household income <10,000 USD v >30,000 USD OR 2.63 (95% CI: 0.98-7.05) (p-value NR)  Seroprevalence in participants with annual household income not declared v >30,000 USD OR 1.15 (95% CI: 0.37-3.57) (p-value NR) |
| [38] | Fiji | September-December 2013 | General population | 2152 participants  417 seropositive | Seroprevalence | None | **Seroprevalence in participants in communities with poverty rate >=40% v <40% poverty rate OR 2.08 (95% CI: 1.67-2.58) (p-value NR)** |
| [38] | Fiji | September-December 2013 | General population | 2152 participants  417 seropositive | Seroprevalence | Gender, ethnicity, community type, metered water available at home, work location | **Seroprevalence in participants in communities with poverty rate >=40% v <40% poverty rate OR 1.74 (95% CI: 1.31-2.31, p<0.001)** |
| [43] | Fiji | 2013 | General population | 2046 participants (number seropositive NR) | Seroprevalence | Cattle density, maximum rainfall in wettest month, distance to river, rural residential setting | **Significantly higher seroprevalence if community poverty rate >40% v <40% poverty rate using Logistic Regression model (p-value NR)** |
| **Education** |  |  |  |  |  |  |  |
| [26] | Federated States of Micronesia | June-September 2011 | Hospital patients | 54 tested  11 confirmed cases | Incidence (confirmed cases only) | None | Cases in participants with education level below secondary v higher than secondary OR 1.0 (95% CI: 0.3-3.9, p=1.000) |
| [35] | American Samoa | May-July 2010 | General population | 807 participants (number seropositive NR) | Seroprevalence | May include male, occupational group, house below median altitude of village, vegetation type, soil type and piggeries within 250 metres and above house | **Seroprevalence in participants who had heard of leptospirosis v never heard of leptospirosis OR 0.60 (95% CI: 0.38-0.96) (p-value NR)** |
| [36] | American Samoa | May-July 2010 | General population | 807 participants  125 seropositive | Seroprevalence | None | **Seroprevalence in participants who had heard of leptospirosis v never heard of leptospirosis OR 0.61 (95% CI: 0.40-0.93) (p-value NR)** |
| [46] | New Zealand | May-October 2013 | Beef, sheep and deer farmers | 178 participants  12 seropositive | Seroprevalence | None | Association between whether participants were aware of leptospirosis and seroprevalence reported NS (p-value NR) |

Note: statistically significant findings (defined by p<0.05 or if p-value not reported, then significance as reported by authors) presented in bold

Abbreviations: CI, confidence interval; NS, not significant; NR, not reported; OR, odds ratio; RR, relative risk

**Table S3:** Findings related to occupational drivers of human *Leptospira* infection

| **Citation details** | **Study location** | **Reported Study period** | **Study Population** | **Sample Size** | **Leptospirosis outcome(s)** | **Covariates in model** | **Findings** |
| --- | --- | --- | --- | --- | --- | --- | --- |
| **Occupation and time worked in that occupation** |  |  |  |  |  |  |  |
| [25] | New Caledonia | January 1985-December 1986 | General population | Total population 145368  193 cases | Incidence; seroprevalence | None | **Farmers-breeders more affected v other occupations χ^2^(1) =13.50 (p<0.05)** |
| [14] (Study 4) | New Caledonia (Coulée) | January 1985-December 1986 | General population | 93 sampled  16 seropositive | Seroprevalence | None | Seroprevalence NS between high-risk occupations (e.g., farmers-breeders, veterinarians) and others χ^2^(1) =1.50 (p>0.20) |
| [26] | Federated States of Micronesia | June-September 2011 | Hospital patients | 54 tested  11 confirmed cases | Incidence (confirmed cases only) | None | Cases in farmers v occupations other than farmers or students OR 9.4 (95% CI: 0.9-103.3, p=0.067)  **Cases in students v occupations other than farmers or students OR 17.5 (95% CI: 1.9-161.1, p=0.012)**  **Cases in students (primary to tertiary) v farming and other occupations OR 17.5 (95% CI: 1.9-161.1, p=0.012)** |
| [29] | New Zealand | November 2009-March 2010 | Sheep abattoir workers | 567 participants  62 seropositive | Seroprevalence | Work position, gender, meat plant | **Years worked at meat plant (continuous) OR 1.1 (95% CI: 1.0-1.1, p=0.011)** |
| [30] | New Zealand | NR | Abattoir workers | 384 sampled  49 seropositive | Incidence | None | New infection in workers who had worked 72-120 at current abattoir v <72 months at current abattoir RR 1.9 (95% CI: 0.7-5.2, p=0.198)  New infection in workers who had worked 120-216 at current abattoir v <72 months at current abattoir RR 1.2 (95% CI: 0.4-3.9, p=0.704)  New infection in workers who had worked >216 months at current abattoir v < 72 months at current abattoir RR 1.3 (95% CI: 0.5-3.2, p=0.558) |
| [30] | New Zealand | NR | Abattoir workers | 384 sampled  49 seropositive | Incidence | None | New infection in workers who had worked 84-198 months in meat industry v <=84 months in meat industry RR 2.6 (95% CI: 1.0-6.9, p=0.059)  New infection in workers who had worked 198-324 months in meat industry v <=84 months in meat industry RR 1.4 (95% CI: 0.5-4.1, p=0.511)  **New infection in workers who had worked >324 months in meat industry v <=84 months in meat industry RR 4.2 (95% CI: 1.7-10.3, p=0.002)** |
| [30] | New Zealand | NR | Abattoir workers | 384 sampled  49 seropositive | Incidence | Work position, abattoir number | **New infection in workers who had worked 72-180 months in meat industry v <=72 months in meat industry RR 3.0 (95% CI: 1.1-7.9, p=0.032)**  New infection in workers who had worked 180-324 months in meat industry v <=72 months in meat industry RR 1.3 (95% CI: 0.4-3.9, p=0.643)  **New infection in workers who had worked >324 months in meat industry v <=72 months in meat industry RR 3.0 (95% CI: 1.1-7.9, p=0.026)** |
| [44] | Vanuatu | January 2013-August 2014 | Hospital patients | 161 patients  12 cases (5 confirmed, 7 probable)  29 seropositive | Incidence (confirmed or probable cases), Seroprevalence | None | Cases in occupations at risk (agriculture, gardening, farming, building, butcher) v occupations not at risk OR 1.51 (95% CI: 0.57-4.01, p=0.41) |
| [16] | New Zealand | February 2008-May 2011 | Sheep abattoir workers | 567 participants, 384 after follow-up (cases NR) | Incidence | Work position, abattoir plant, gender | Months worked in meat industry (continuous) OR 1.0 (95% CI: 0.9-1.0, p=0.279)  Cases in participants who owned a farm with pigs, goats, sheep, beef cattle, alpaca or deer v other participants OR 0.6 (95% CI: 0.2-1.7, p=0.392) |
| [46] | New Zealand | May-October 2013 | Beef, sheep and deer farmers | 178 participants  12 seropositive | Seroprevalence | None | Years farming (continuous) reported NS (p-value NR) |
| [49] | New Zealand | Not reported | Pig farmers | 70 participants  20 seropositive | Seroprevalence | None | Part-time v full-time pig farmer reported NS (p-value NR)  Farming more than 10 years v farming less than 10 years reported NS (p-value NR) |
| [51] | New Zealand | 1990-1998 | Notification data | Total population NR  1397 cases | Incidence | None | **Cases in livestock** **farm workers v workers other than livestock, meat or forestry workers RR 91.2 (95% CI: 75.2-110.6) (p-value NR)**  **Cases in meat processing workers v workers other than livestock, meat or forestry workers RR 163.5 (95% CI: 133.1-200.1) (p-value NR)**  **Cases in forestry workers v workers other than livestock, meat or forestry workers RR 24.0 (95% CI: 14.5-39.7) (p-value NR)**  **Significantly different serovars between main occupational groups χ^2^(9) =1.50 (p<0.0001)** |
| **Indoor v outdoor occupation** |  |  |  |  |  |  |  |
| [35] | American Samoa | May-July 2010 | General population | 807 participants (number seropositive NR) | Seroprevalence | May include male, house below median altitude of village, vegetation type, soil type, piggeries within 250 metres and above house | **Seroprevalence in outdoor occupations (including fish cleaners) v indoor occupations** **OR 2.77 (95% CI: 1.40-5.49) (p-value NR)**  Seroprevalence in mixed indoor/outdoor occupations v indoor occupations OR 1.14 (95% CI: 0.46-2.87) (p-value NR)  Seroprevalence in unemployed participants v indoor occupations OR 1.59 (95% CI: 0.85-2.98) (p-value NR) |
| [36] | American Samoa | May-July 2010 | General population | 807 participants  125 seropositive | Seroprevalence | None | **Seroprevalence in outdoor occupations v indoor occupations OR 3.25 (95% CI: 1.79-5.90) (p-value NR)**  Seroprevalence in mixed indoor/outdoor occupations v indoor occupations OR 1.39 (95% CI: 0.62-3.13) (p-value NR)  Seroprevalence in unemployed participants v indoor occupations OR 1.51 (95% CI: 0.88-2.59) (p-value NR) |
| [36] | American Samoa | May-July 2010 | General population | 807 participants  125 seropositive | Seroprevalence | Male, heard of leptospirosis, occupation, piggeries within 250 m and above house | **Seroprevalence in outdoor occupations v indoor occupations OR 2.69 (95% CI: 1.43-5.06) (p-value NR)**  Seroprevalence in mixed indoor/outdoor occupations v indoor occupations OR 0.82 (95% CI: 0.36-1.91) (p-value NR)  Seroprevalence in unemployed participants v indoor occupations OR 1.62 (95% CI: 0.92-2.86) (p-value NR) |
| [38] | Fiji | September-December 2013 | General population | 2152 participants  417 seropositive | Seroprevalence | None | **Seroprevalence in mixed indoor/outdoor occupations v indoor occupations OR 1.63 (95% CI: 1.23-2.17) (p-value NR)**  **Seroprevalence in outdoor occupations v indoor occupations OR 2.59 (95% CI: 1.93-3.47) (p-value NR)** |
| [38] | Fiji | September-December 2013 | General population | 2152 participants  417 seropositive | Seroprevalence | Gender, ethnicity, community type, metered water available at home, work location | **Seroprevalence in mixed indoor/outdoor occupations v indoor occupations OR 1.65 (95%: CI 1.23-2.20, p=0.001)**  **Seroprevalence in outdoors v indoor occupations OR 1.64 (95% CI: 1.15-2.34, p=0.006)** |
| **Meat worker-specific factors** |  |  |  |  |  |  |  |
| [21] | New Zealand | July-November 1978 | Meat inspectors | 1003 sampled  103 seropositive | Seroprevalence | None | **Higher seroprevalence in workers at pig works v other meat works (p<0.001)** |
| [22] | New Zealand | 1979-1980 | Meat inspectors and workers | Inspectors: 1215 sampled, 121 seropositive  Workers: 1248 sampled, 77 seropositive | Seroprevalence | None | **Hardjo: seroprevalence for work with live animals, carcasses, edible viscera v other work (p<0.0001)**  **Pomona: seroprevalence for work with live animals, carcasses, edible viscera v other work (p<0.0001)**  **Tarassovi: seroprevalence for work with live animals, carcasses, edible viscera v other work (p<0.0001)**  Copenhageni: seroprevalence for work with live animals, carcasses, edible viscera v other work reported NS (p-value NR)  Ballum: seroprevalence for work with live animals, carcasses, edible viscera v other work reported NS (p-value NR)  **Seroprevalence for workers on slaughter floor v other work with live animals, carcasses and edible viscera (p<0.0001)**  **Seroprevalence for meat workers v meat inspectors (p<0.001)**  Seroprevalence for slaughterboard workers v meat inspectors reported NS (p-value NR)  **Seroprevalence for contact with pigs at works v contact with other stock (p<0.0001)**  **Seroprevalence based on years in contact with pigs at works v contact with other stock (p<0.01)**  **Increased seroprevalence for workers processing pigs only (F) v other works (A-E) (p<0.0001)** |
| [15] | New Zealand | February 2008-May 2011 | Abattoir workers | 592 participants  51 new infections | Incidence | None | **Higher annual infection risk in sheep workers v beef workers (p<0.001)**  **Higher annual infection risk in sheep workers v deer workers (p=0.01)** |
| [29] | New Zealand | November 2009-March 2010 | Sheep abattoir workers | 567 participants  62 seropositive | Seroprevalence | None | **Seroprevalence for workers in offal, pet food v boning, chillers, office OR 5.8 (95% CI: 1.3-25.2, p=0.02)**  **Seroprevalence for workers in gut & kidney removal, meat inspection v boning, chillers, office OR 9.6 (95% CI: 2.5-36.3, p=0.001)**  **Seroprevalence for workers in yards, stunning, pelting v boning, chillers, office OR 17.4 (95% CI: 5.0-60.0, p<0.001)**  Deer workers:  **Seroprevalence for workers in offal, pet food, gut & kidney removal, yards, stunning, pelting v boning, chillers, office OR 21.2 (95% CI: 2.4-183.7, p=0.006)**  Beef workers:  Seroprevalence for workers in maintenance v boning, chillers, office OR 3.0 (95% CI: 0.3-31.6, p=0.368)  Seroprevalence for workers in offal, pet food v boning, chillers, office OR 2.8 (95% CI: 0.4-18.0, p=0.276)  Seroprevalence for workers in yards, stunning, pelting, gut & kidney removal, meat inspection v boning, chillers, office OR 1.6 (95% CI: 0.3-7.4, p=0.552) |
| [29] | New Zealand | November 2009-March 2010 | Abattoir workers | 567 participants  62 seropositive | Seroprevalence | Gender, years worked at meat plant, meat plant (sheep workers); Personal Protective Equipment (deer workers); age in years (beef workers) | Sheep workers:  **Seroprevalence for workers in offal, pet food v boning, chillers, office OR 6.5 (95% CI: 1.4-29.8, p=0.017)**  **Seroprevalence for workers in gut removal, pulling kidneys v boning, chillers, office OR 8.2 (95% CI: 2.1-32.7, p=0.003)**  **Seroprevalence for workers in yards, stunning, pelting v boning, chillers, office OR 10.4 (95% CI: 2.8-38.8, p<0.001)**  Deer workers:  **Seroprevalence for workers in offal, pet food, gut removal, pulling kidneys, yards, stunning, pelting v boning, chillers, office OR 12.7 (95% CI: 1.3-120.6, p=0.027)**  Beef workers:  Seroprevalence for workers in maintenance v boning, chillers, office OR 2.0 (95% CI 0.3-23.4, p=0.59)  Seroprevalence for workers in offal, pet food v boning, chillers, office OR 3.1 (95% CI 0.5-20.6, p=0.25)  Seroprevalence for workers in yards, stunning, pelting, gut, kidney removal, meat inspection v boning, chillers, office OR 2.2 (95% CI 0.5-10.8, p=0.32) |
| [29] | New Zealand | November 2009-March 2010 | Sheep abattoir workers | 567 participants  62 seropositive | Seroprevalence | None | Seroprevalence in workers at sheep plant 2 v sheep plant 1 OR 1.0 (95% CI: 0.4-2.3, p=0.965)  **Seroprevalence in workers at** **sheep plant 3 v sheep plant 1 OR 3.5 (95% CI: 1.3-9.1, p=0.011)**  Seroprevalence in workers at sheep plant 4 v sheep plant 1 OR 0.8 (95% CI: 0.3-2.1, p=0.692) |
| [29] | New Zealand | November 2009-March 2010 | Sheep abattoir workers | 567 participants  62 seropositive | Seroprevalence | Work position, gender, years worked at meat plant | Seroprevalence in workers at sheep plant 2 v sheep plant 1 OR 4.5 (95% CI: 1.2-16.3, p=0.022)  **Seroprevalence in workers at** **sheep plant 3 v sheep plant 1 OR 6.3 (95% CI: 1.8-22.4, p=0.004)** Seroprevalence in workers at sheep plant 4 v sheep plant 1 OR 2.1 (95% CI: 0.7-6.3, p=0.201) |
| [30] | New Zealand | NR | Abattoir workers | 384 sampled  49 seropositive | Incidence | None | Seroprevalence for workers in offal, pet food v boning, chillers, office RR 3.2 (95% CI: 0.8-12.9, p=0.098)  **Seroprevalence for workers in gut & kidney removal, meat inspection v boning, chillers, office RR 5.2 (95% CI: 1.7-16.1, p=0.004)**  **Seroprevalence for workers in yards, stunning, hide removal v boning, chillers, office RR 8.4 (95% CI 2.9-24, p<0.01)** |
| [30] | New Zealand | NR | Abattoir workers | 384 sampled  49 seropositive | Incidence | Months worked in meat industry, abattoir number | **Seroprevalence for workers in offal, pet food v boning, chillers, office** **RR 4.1 (95% CI: 1.0-16.4, p=0.048)**  **Seroprevalence for workers in gut, meat inspection, pulling kidneys v boning, chillers, office RR 5.2 (95% CI: 1.7-16.0, p=0.004)**  **Seroprevalence for workers in yards, stunning, pelting adjusted v boning, chillers, office RR 7.5 (95% CI: 2.5-22.4, p<0.001)** |
| [30]* | New Zealand | NR | Abattoir workers | 384 sampled  49 seropositive | Incidence | None | Seroprevalence for workers in sheep plant 1 (sample 2) v sheep plant 1 (sample 1) RR 0.7 (95% CI: 0.3-1.6, p=0.418)  Seroprevalence for workers in sheep plant 2 v sheep plant 1 (sample 1) RR 1.3 (95% CI: 0.6-3.0, p=0.511)  Seroprevalence for workers in sheep plant 3 v sheep plant 1 (sample 1) RR 1.1 (95% CI: 0.3-3.8, p=0.923)  Seroprevalence for workers in sheep plant 4 v sheep plant 1 (sample 1) RR 0.8 (95% CI: 0.3-1.9, p=0.563) |
| [30]* | New Zealand | NR | Abattoir workers | 384 sampled  49 seropositive | Incidence | Work position, months worked in meat industry | **Seroprevalence for workers in sheep plant 2 v sheep plant 1 (sample 1), sheep plant 3, sheep plant 4, sheep plant 1 (sample 2)** **OR 2.0 (95% CI: 1.0-3.9, p=0.046)** |
| [16] | New Zealand | February 2008-May 2011 | Sheep abattoir workers | 567 participants, 384 after follow-up (cases NR) | Incidence | None | Generalised Linear Modelling:  **New infection for workers in offal removal, pet food v not working in offal removal, pet food OR 14.1 (95% CI: 2.0-280.0, p=0.019)**  **New infection for workers removing intestines or kidneys, meat inspection v not removing intestines or kidneys, not inspecting meat OR 22.3 (95% CI: 4.3-409.5, p=0.003)**  **New infection for workers in yards, stunning or pelting v not working in yards, not stunning or pelting OR 29.4 (95% CI: 6.0-533.5, p=0.001)** |
| [16] | New Zealand | February 2008-May 2011 | Sheep abattoir workers | 567 participants, 384 after follow-up (cases NR) | Incidence | Abattoir plant, gender | Generalised Linear Modelling:  **New infection for** **workers in offal removal, pet food v not working in offal removal, pet food OR 22.1 (95% CI: 2.3-209.8) (p-value NR)**  **New infection for** **workers in intestines or kidney removal, meat inspection v not removing intestines or kidneys, not inspecting meat OR 33.8 (95% CI: 4.2-271.1) (p-value NR)**  **New infection for** **workers in yards, stunning or pelting v not working in yards, not stunning or pelting adjusted OR 57.0 (95% CI: 6.9-473.3) (p-value NR)**  Additive Bayesian Network:  **New infection for** **workers in offal removal, pet food v not working in offal removal, pet food OR 18.3 (95% CI: 2.2-506.7) (p-value NR)**  **New infection for** **workers in intestines or kidney removal, meat inspection v not removing intestines or kidneys, not inspecting meat OR 30.7 (95% CI: 4.9-788.4) (p-value NR)**  **New infection for** **workers in yards, stunning or pelting v not working in yards, not stunning or pelting adjusted OR 41.0 (95% CI: 6.9-1044.2) (p-value NR)** |
| [16]* | New Zealand | February 2008-May 2011 | Sheep abattoir workers | 567 participants, 384 after follow-up (cases NR) | Incidence | None | New infection for workers in abattoir plant 1 (sample 2) v not working in abattoir plant 1 (sample 2) OR 2.3 (95% CI: 0.8-7.3, p=0.132)  **New infection for workers in abattoir plant 2 v not working in abattoir plant 2 OR 4.6 (95% CI: 1.7-13.8, p=0.004)**  New infection for workers in abattoir plant 3 v not working in abattoir plant 3 OR 2.3 (95% CI: 0.3-10.7, p=0.338)  New infection for workers in abattoir plant 4 v not working in abattoir plant 4 OR 2.5 (95% CI: 0.8-7.7, p=0.108) |
| [16] | New Zealand | February 2008-May 2011 | Sheep abattoir workers | 567 participants, 384 after follow-up (cases NR) | Incidence | Work position, gender | Generalised Linear Modelling:  **New infection for workers in abattoir plant 2 v not working in abattoir plant 2 OR 4.5 (95% CI: 1.9-10.7) (p-value NR)**  **New infection for workers in abattoir plant 4 v not working in abattoir plant 4 OR 3.4 (95% CI: 1.3-8.9) (p-value NR)** |
| **Animal urine contact** |  |  |  |  |  |  |  |
| [30] | New Zealand | NR | Abattoir workers | 384 sampled  49 seropositive | Incidence | None | Cases in workers who said yes to urine splashed in face v workers who said no to urine splashed in face RR 0.9 (95% CI: 0.4-1.7, p=0.664)  Cases in workers who said don't know/maybe to urine splashed in face v workers who said no to urine splashed in face OR 1.5 (95%: CI 0.2-10.9, p=0.695) |
| [46] | New Zealand | May-October 2013 | Beef, sheep and deer farmers | 178 participants  12 seropositive | Seroprevalence | None | Association between animal urine exposure and seroprevalence reported as NS (p-value NR) |
| **Farm-worker specific factors** |  |  |  |  |  |  |  |
| [23] | New Zealand | Not reported | Dairy farm workers | 308 sampled  137 seropositive | Seroprevalence | None | Hardjo: Seroprevalence in participants with previous clinical history of leptospirosis in herd v no previous clinical history of leptospirosis in herd reported NS (p-value NR)  **Pomona: Seroprevalence in participants with previous clinical history of leptospirosis in herd v no clinical history of leptospirosis in herd (p<0.01)**  **Hardjo or Pomona: Seroprevalence in workers who vaccinated their herd v those who did not vaccinate their herd (p<0.05)**  Both serovars: Seroprevalence in workers who vaccinated their herd v those who did not vaccinate their herd reported NS (p-value NR)  Hardjo or Pomona: Association between herd size and seroprevalence in workers reported NS (p-value NR)  **Both serovars: Seroprevalence in workers based on herd size (p<0.05)**  **Higher seroprevalence to Hardjo in workers with less time milking v more time milking (p<0.05)**  **Higher seroprevalence to Hardjo in workers with herringbone and rotary sheds v walk-through sheds (p<0.05)**  Seroprevalence in workers who buy in stock v workers who did not buy in stock reported NS (p-value NR)  Seroprevalence in workers keeping pigs v others reported NS (p-value NR)  Seroprevalence in workers docking cow’s tails v workers who did not dock cow’s tails reported NS (p-value NR)  Seroprevalence in workers producing milk for factory supply v workers producing milk for town supply reported NS (p-value NR) |
| [40] | New Zealand | Not reported | Dairy farm workers | 226 participants, 213 sampled  84 seropositive | Seroprevalence | None | Hardjo, Pomona or both: seroprevalence in workers with clinical history of leptospirosis in herd v no clinical history of leptospirosis in herd reported NS (p-value NR)  Hardjo: seroprevalence for workers keeping pigs for home consumption v for sale reported NS; keeping pigs for sale v none reported NS (p-value NR)  **Pomona: seroprevalence for workers keeping pigs for home consumption v for sale (p<0.05); keeping pigs for sale v none (p<0.05)**  Both serovars: seroprevalence for workers keeping pigs for home consumption v for sale reported NS; keeping pigs for sale v none reported NS (p-value NR)  **Hardjo: seroprevalence for milkers v non-milkers (p<0.05)**  **Both serovars: seroprevalence for milkers v non-milkers (p<0.01)**  **Hardjo: seroprevalence for workers spending up to 2 hours in milking shed v 2 or more hours (p<0.05)**  Hardjo, Pomona or both: seroprevalence workers with Herringbone plus rotary shed v walkthrough reported NS (p-value NR)  Hardjo, Pomona or both: seroprevalence workers using milk for factory supply v town supply reported NS (p-value NR)  Seroprevalence for workers participating in udder washing or teat stimulation v not participating in udder washing or teat stimulation reported NS (p-value NR) |
| [17] | New Zealand | Not reported | Dairy farm workers | 25 cases farms  27 control farms (number of individuals not reported) | Seroprevalence | None | Titres to Hardjo >=1:24 in cattle in high-risk farms (one or more seropositive workers) v control farms (no seropositive workers) (p=0.05, authors reported as significant)  **Geometric mean titre of Hardjo in cattle in high-risk farms v control farms (p=0.01)**  Number of cows in high-risk farms v control farms NS (p=0.27)  Number of breeding cattle and/or breeding sows in high-risk farms v control farms reported NS (p-value NR) |
| [46] | New Zealand | May-October 2013 | Beef, sheep and deer farmers | 178 participants  12 seropositive | Seroprevalence | None | **Seroprevalence in participants farming deer alone or with beef/sheep v farming beef and/or sheep coefficient 1.06 (95% PI: -0.12-2.52, p=0.03)** |
| [46] | New Zealand | May-October 2013 | Beef, sheep and deer farmers | 178 participants  12 seropositive | Seroprevalence | Assisting calving/fawning, wild deer abundance on farm, flat terrain on farm, possum abundance on farm | **Seroprevalence in participants farming deer alone or with beef/sheep v farming beef and/or sheep coefficient 1.99 (median prevalence ratio 6.9, 95% PI: 1.6-40.8) (p-value NR)** |
| [46] | New Zealand | May-October 2013 | Beef, sheep and deer farmers | 178 participants  12 seropositive | Seroprevalence | None | **Seroprevalence in participants assisting calving/fawning v not assisting calving/fawning coefficient 1.24 (95% PI: -0.07-2.86, p=0.03)** |
| [46] | New Zealand | May-October 2013 | Beef, sheep and deer farmers | 178 participants  12 seropositive | Seroprevalence | Wild deer abundance on farm, species farmed, flat terrain on farm, possum abundance on farm | **Seroprevalence in participants assisting calving/fawning v not assisting calving/fawning coefficient 2.02 (median prevalence ratio 7.2, 95% PI: 1.7-42.7) (p-value NR)** |
| [46] | New Zealand | May-October 2013 | Beef, sheep and deer farmers | 178 participants  12 seropositive | Seroprevalence | None | Seroprevalence in participants with flat terrain of farm >=25% v flat terrain of farm <25% coefficient 0.76 (95% PI: -0.46-2.14, p=0.11) |
| [46] | New Zealand | May-October 2013 | Beef, sheep and deer farmers | 178 participants  12 seropositive | Seroprevalence | Assisting calving/fawning, wild deer abundance on farm, species farmed, possum abundance on farm | **Seroprevalence in participants with flat terrain of farm >=25% v flat terrain of farm <25% coefficient** **1.47 (median prevalence ratio 4.2, 95% PI: 1.1-20.9) (p-value NR)** |
| [46] | New Zealand | May-October 2013 | Beef, sheep and deer farmers | 178 participants  12 seropositive | Seroprevalence | None | Pomona prevalence beef (continuous) coefficient 1.78 (95% PI: -7.12-1.85, p=0.19); Leptospira prevalence beef (continuous) and Hardjo prevalence beef (continuous) reported NS (p-value NR)  Pomona prevalence deer (continuous) coefficient -3.02 (95% PI: -11.78-2.63, p=0.17); Leptospira prevalence deer (continuous) reported NS (p-value NR)  Pomona, Hardjo or Leptospira prevalence sheep (continuous) reported NS (p-value NR)  Number of beef x 100 animals (continuous) coefficient 0.09 (95% PI: -0.31-0.06, p=0.12)  **Number of sheep x 100 animals (continuous) coefficient 0.03 (95% PI: -0.06 to -0.01, p=0.01)**  Number of deer x 100 animals (continuous) reported NS (p-value NR)  **Valley pond water source coefficient -1.46 (95% PI: -3.08 to -0.15, p=0.02)**  Other drivers of infection reported NS for association with seroprevalence in participants: effluent from farm into oxidation ponds vs pastures, flooding of farmland, farm next to bush or forest, assisting lambing, crutching dagging sheep, shearing sheep, cleaning urine from yards, milking cows, castrating lambs, castrating calves (p-value NR) |
| [49] | New Zealand | Not reported | Pig farmers | 70 participants  20 seropositive | Seroprevalence | None | Seroprevalence in farmers with number of breeding sows greater than 50 v less than 50 (p=0.05, authors reported as significant)  Seroprevalence in farmers with number of fattening pigs 100-150 significant v less than 100 (p=0.05, authors reported as significant)  Seroprevalence in farmers with number of fattening pigs v less than 100 more than 500 significant (p=0.05, authors reported as significant)  Seroprevalence in farmers with stock always inside v sows at pasture (p=0.05, authors reported as significant)  Seroprevalence in farmers buying in stock v others reported NS (p-value NR)  Seroprevalence in farmers with clinical history of leptospirosis in herd v farmers without clinical history of leptospirosis in herd reported NS (p-value NR)  Seroprevalence in farmers who vaccinated stock against leptospirosis v stock not vaccinated reported NS (p-value NR)  Seroprevalence in farmers with effluent from farm into oxidation ponds v pastures reported NS (p-value NR) |
| **Personal Protective Equipment** |  |  |  |  |  |  |  |
| [23] | New Zealand | Not reported | Dairy farm workers | 308 sampled  137 seropositive | Seroprevalence | None | Seroprevalence in workers wearing glasses and protective clothing v not wearing glasses and protective clothing reported NS (p-value NR) |
| [29] | New Zealand | November 2009-March 2010 | Abattoir workers | 567 participants  62 seropositive | Seroprevalence | None | Sheep workers:  Seroprevalence in workers always wearing gloves on both hands v never OR 0.9 (95% CI: 0.4-1.9, p=0.806)  Seroprevalence in workers often wearing gloves on both hands v never OR 0.8 (95% CI: 0.1-6.8, p=0.829)  Seroprevalence in workers sometimes wearing gloves on both hands v never OR 0.9 (95% CI: 0.3-2.8, p=0.894)  **Seroprevalence in workers always wearing safety glasses v never OR 2.5 (95% CI: 1.1-5.6, p=0.028)**  Seroprevalence in workers often wearing safety glasses v never OR 2.5 (95% CI: 0.5-9.1, p=0.261)  Seroprevalence in workers sometimes wearing safety glasses v never OR 2.5 (95% CI: 0.9-7.3, p=0.085)  **Seroprevalence in workers often or always wearing a facemask v never or sometimes OR 2.8 (95% CI 1.0-7.6, p=0.04)**  Seroprevalence in workers often or always wearing a balaclava never or sometimes OR 1.7 (95% CI 0.9-3.2, p=0.1)  Deer workers:  Seroprevalence in workers always wearing gloves on both hands v never OR 0.6 (95% CI: 0.1-3.6, p=0.624)  Seroprevalence in workers sometimes wearing gloves on both hands v never OR 0.4 (95% CI: 0.0-3.7, p=0.416)  **Seroprevalence in workers always wearing safety glasses v never OR 13.0 (95% CI: 2.5-66.4, p=0.002)**  Seroprevalence in workers sometimes wearing safety glasses v never OR 6.5 (95% CI: 0.4-94.1, p=0.17)  Seroprevalence in workers often or always wearing a facemask v never or sometimes OR 5.1 (95% CI 0.3-89.5, p=0.264)  Seroprevalence in workers often or always wearing a balaclava never or sometimes OR 1.2 (95% CI 0.1-12.0, p=0.88)  Beef workers:  Seroprevalence in workers always wearing gloves on both hands v never OR 2.8 (95% CI: 0.4-24.3, p=0.309)  Seroprevalence in workers always wearing safety glasses v never OR 0.7 (95% CI: 0.1-3.4, p=0.644)  Seroprevalence in workers often wearing safety glasses v never OR 4.1 (95% CI: 0.4-41.8, p=0.233)  Seroprevalence in workers often or always wearing a facemask v never or sometimes OR 6.4 (95% CI: 0.6-67.5, p=0.124) |
| [29] | New Zealand | November 2009-March 2010 | Deer abattoir workers | 567 participants  62 seropositive | Seroprevalence | Work position | Seroprevalence in workers often or always wearing facemask or safety goggles v never or sometimes OR 4.3 (95% CI: 0.8-22.8, p=0.093) |
| [30] | New Zealand | NR | Abattoir workers | 384 sampled  49 seropositive | Incidence | None | Sheep workers:  Seroprevalence in workers wearing gloves on both hands always v never RR 1.4 (95% CI: 0.7-2.9, p=0.307)  Seroprevalence in workers wearing gloves on both hands often v never RR 2.3 (95% CI: 0.7-7.4, p=0.152)  Seroprevalence in workers wearing gloves on both hands sometimes v never RR 0.4 (95% CI: 0.0-2.8, p=0.333)  Seroprevalence in workers wearing goggles/glasses always v never RR 1.8 (95% CI: 0.9-3.5, p=0.077)  Seroprevalence in workers wearing goggles/glasses often v never RR 0.9 (95% CI: 0.1-6.9, p=0.917)  Seroprevalence in workers wearing goggles/glasses sometimes v never RR 0.9 (95% CI: 0.2-4.0, p=0.887)  Seroprevalence in workers wearing a facemask often or always v never RR 1.0 (95% CI: 0.5-2.2, p=0.948)  Seroprevalence in workers wearing a balaclava often or always v never RR 1.2 (95% CI: 0.6-2.2, p=0.617) |
| [40] | New Zealand | Not reported | Dairy farm workers | 226 participants, 213 sampled  84 seropositive | Seroprevalence | None | **Both serovars: seroprevalence in workers wearing shorts in shed v workers not wearing shorts in shed (p<0.05)**  Hardjo or Pomona: seroprevalence in workers wearing shorts in shed v workers not wearing shorts reported NS (p-value NR)  **Hardjo: seroprevalence in workers wearing apron in shed v not wearing apron in shed (p<0.05)**  Pomona or both serovars: wearing apron in shed v not wearing apron in shed reported NS (p-value NR) |
| [16] | New Zealand | February 2008-May 2011 | Sheep abattoir workers | 567 participants, 384 after follow-up (cases NR) | Incidence | None | Cases in workers wearing normal or safety glasses always/often v sometimes/never OR 2.1 (95% CI: 1.0-4.7, p=0.053)  Cases in workers wearing gloves on both hands always/often v sometimes/never OR 2.0 (95% CI: 0.9-4.8, p=0.099)  Cases in workers wearing a facemask always/often v sometimes/never OR 1.2 (95% 0.5-2.8, p=0.639) |
| [49] | New Zealand | Not reported | Pig farmers | 70 participants  20 seropositive | Seroprevalence | None | Association between seroprevalence and type of protective clothing worn by farmer reported NS (p-value NR) |

*Sheep worker abattoir plant 1 took part in the study in two consecutive years; 57/160 (35.6%) persons were sampled twice

Note: statistically significant findings (defined by p<0.05 or if p-value not reported, then significance as reported by authors) presented in bold

Abbreviations: CI, confidence interval; NS, not significant; NR, not reported; OR, odds ratio; PI, probability interval; RR, relative risk

**Table S4:** Findings related to lifestyle drivers of human *Leptospira* infection

| **Citation details** | **Study location** | **Reported Study period** | **Study Population** | **Sample Size** | **Leptospirosis outcome** | **Covariates in model** | **Findings** |
| --- | --- | --- | --- | --- | --- | --- | --- |
| **Water-associated exposures** |  |  |  |  |  |  |  |
| [20] | Palau, Guam, Federated States of Micronesia, Vanuatu, Fiji, Tonga, Wallis, Futuna and French Polynesia | September 2003-December 2005 | Hospital patients | 263 suspected  69 confirmed cases | Incidence (confirmed cases only) | None | Case frequency in participants bathing in freshwater v not bathing in freshwater reported NS at region or island level (p-value NR)  Case frequency in participants fishing v not fishing reported NS at region or island level (p-value NR) |
| [14] (Study 2) | New Caledonia (Nera) | January 1985-December 1986 | General population | 41 sampled  16 seropositive | Seroprevalence | None | **Difference in number of participants with frequent and repeated contact with water was significant between cases v seronegatives χ^2^(1)=5.43 (p<0.05)** |
| [14] (Study 4) | New Caledonia (Coulée) | January 1985-December 1986 | General population | 93 sampled  16 seropositive | Seroprevalence | None | **Frequency of contact with fresh water is higher in seropositives v seronegatives χ^2^(1)=4.27 (p<0.05)**  **Seropositive participants had contact with water more frequently than seronegatives χ^2^(1)=4.36 (p<0.05)** |
| [26] | Federated States of Micronesia | June-September 2011 | Hospital patients | 54 tested  11 confirmed cases | Incidence (confirmed cases only) | None | Case frequency in participants drinking from stream v others OR 1.7 (95% CI: 0.4-6.4, p=0.496)  Cases frequency in participants bathing from stream v others OR 1.2 (95% CI: 0.3-4.7, p=1.000)  Cases frequency in participants who swim or stand in freshwater v others OR 3.8 (95% CI: 0.9-15.2, p=0.081) |
| [27] | French Polynesia | March 2004-March 2005 | Hospital patients | 113 participants  33 cases (22 confirmed, 11 probable) | Incidence (confirmed or probable) | None | **Swimming in river more frequent in confirmed/suspected cases v unconfirmed cases (p<0.02 overall, p<0.01 in Raiatea)**  Fishing in confirmed/suspected cases v unconfirmed cases reported NS (p-value NR) |
| [32] | New Caledonia | January-June 2008 | Hospital patients | 135 cases (101 confirmed, 34 probable)  For inferential statistics: 98 cases, 410 controls | Incidence (confirmed or probable) | None | **Freshwater swimming in confirmed cases v negative participants OR 3.19 (95% CI: 1.96-5.19) (p-value NR)**  **Freshwater fishing in confirmed cases v negative participants OR 3.00 (95% CI: 1.68-5.37) (p-value NR)** |
| [36] | American Samoa | May-July 2010 | General population | 807 participants  125 seropositive | Seroprevalence | None | **Seropositive participants swimming at beach frequently (>1 time per week) v never OR 2.01 (95% CI 1.23-3.26) (p-value NR)**  Seropositive participants swimming at beach occasionally (<1 time per week) v never OR 1.20 (95% CI: 0.73-1.96) (p-value NR)  **Seropositive** **participants swimming or walking in rain puddles frequently (>1 time per week) v never OR 1.52 (95% CI: 1.00-2.32) (p-value NR)**  Seropositive participants swimming or walking in rain puddles occasionally (<1 time per week) v never OR 0.83 (95% CI: 0.47-1.49) (p-value NR)  **Seropositive participants fishing frequently (>1 time per week) v never OR 1.78 (95% CI: 1.11-2.83) (p-value NR)**  Seropositive participants fishing occasionally (<1 time per week) v never OR 1.43 (95%: CI 0.82-2.48) (p-value NR) |
| [44] | Vanuatu | January 2013-August 2014 | Hospital patients | 161 patients  12 cases (5 confirmed, 7 probable)  29 seropositive | Seroprevalence | None | **Seropositive participants in contact with surface water v no contact with surface water OR 4.36 (95% CI: 1.71-11.10, p=0.002)** |
| [44] | Vanuatu | January 2013-August 2014 | Hospital patients | 161 patients  12 cases (5 confirmed, 7 probable)  29 seropositive | Seroprevalence | Sex, water at home, freshwater fishing, hunting, contact with animals (pigs, dogs/ cats, horses /goats/ sheep, cattle), rodents seen in vicinity, island of residence, occupational risk | Seropositive participants in contact with surface water v no contact with surface water OR 2.76 (95% CI: 0.83-9.23, p=0.10) |
| [44] | Vanuatu | January 2013-August 2014 | Hospital patients | 161 patients  12 cases (5 confirmed, 7 probable)  29 seropositive | Seroprevalence | None | Seropositive participants fishing in freshwater v no fishing in freshwater OR 2.77 (95% CI: 0.93-8.23, p=0.07) |
| [44] | Vanuatu | January 2013-August 2014 | Hospital patients | 161 patients  12 cases (5 confirmed, 7 probable)  29 seropositive | Seroprevalence | Sex, water at home, contact with surface water, hunting, contact with animals (pigs, dogs/ cats, horses /goats/ sheep, cattle), rodents seen in vicinity, island of residence, occupational risk | Seropositive participants fishing in freshwater v no fishing in freshwater OR 0.73 (95% CI 0.14-3.89, p=0.71) |
| [45] | New Caledonia | 1989 | General population | Total population 165000  144 cases | Incidence | None | NS association reported between serogroups and swimming in river water and fishing (p-value NR) |
| [46] | New Zealand | May-October 2013 | Beef, sheep and deer farmers | 178 participants  12 seropositive | Seroprevalence | None | NS association reported between seroprevalence in farmers and outdoor freshwater exposure, fishing lakes/rivers and water sports lakes/rivers (p-value NR) |
| [47] | New Zealand | May/June 2012 | Veterinarians | 277 participants  14 seropositive | Seroprevalence | None | Seropositive participants fishing v not fishing OR 3.0 (95%: CI 1.0-8.9, Likelihood Ratio Test p=0.06) |
| [48] | Hawaii | July 1988-June 1989 (Big Island); July-December 1988 (Kauai) | Hospital patients | Big Island: 172 participants, 123 followed, 20 seropositive  Kauai: 100 participants, 59 followed, 13 cases  Inferential statistics: 33 cases, 77 controls | Incidence | None | Cases who drank surface water (stream) v controls who drank surface water (stream) (p=0.08) |
| **Non-water associated activities** |  |  |  |  |  |  |  |
| [20] | Palau, Guam, Federated States of Micronesia, Vanuatu, Fiji, Tonga, Wallis, Futuna and French Polynesia | September 2003-December 2005 | Hospital patients | 263 suspected  69 confirmed cases | Incidence (confirmed cases only) | None | Case frequency in participants hunting v not hunting reported NS at regional or island level (p-value NR) |
| [26] | Federated States of Micronesia | June-September 2011 | Hospital patients | 54 tested  11 confirmed cases | Incidence (confirmed cases only) | None | Cases frequency in participants walking through mud v not walking through mud OR NR (p=0.096)  **Cases frequency in participants tending to gardens or crops v not tending to gardens or crops OR 8.6 (95% CI 1.0-73.8, p=0.035)**  Case frequency in participants who reported recent pig slaughter v no recent pig slaughter OR 0.7 (95% CI: 0.8-7.1, p=1.000) |
| [27] | French Polynesia | March 2004-March 2005 | Hospital patients | 113 participants  33 cases (22 confirmed, 11 probable) | Incidence (confirmed or probable) | None | Any serovar: hunting in confirmed/suspected cases v unconfirmed cases reported NS (p-value NR)  **Canicola: hunting reported to be** **significantly associated with Canicola cases (p<0.01)** |
| [30] | New Zealand | NR | Abattoir workers | 384 sampled  49 seropositive | Incidence | None | New infection in workers hunting pigs, deer or feral goats v not hunting pigs, deer or feral goats RR 1.1 (95% CI: 0.4-3.2, p=0.804)  New infection in workers farming pigs, goats, sheep, beef cattle, alpaca or deer v not farming pigs, goats, sheep, beef cattle, alpaca or deer RR 0.8 (95% CI: 0.3-1.8, p=0.53)  New infection in workers slaughtering sheep, goats, pigs, beef or deer at home v no slaughtering sheep, goats, pigs, beef or deer at home RR 0.9 (95% CI: 0.4-2.0, p=0.744) |
| [32] | New Caledonia | January-June 2008 | Hospital patients | 135 cases (101 confirmed, 34 probable)  For inferential statistics: 98 cases, 410 controls | Incidence (confirmed or probable) | None | **Hunting in confirmed cases v negative cases OR 3.33 (95% CI: 1.74-6.36) (p-value NR)** |
| [44] | Vanuatu | January 2013-August 2014 | Hospital patients | 161 patients  12 cases (5 confirmed, 7 probable)  29 seropositive | Incidence (confirmed or probable cases), Seroprevalence | None | Frequency of hunting in seropositive cases v negative OR 0.71 (95% CI: 0.08-6.04, p=0.75) |
| [45] | New Caledonia | 1989 | General population | Total population 165000  144 cases | Incidence | None | Case frequency in participants hunting v not hunting reported NS (p-value NR) |
| [16] | New Zealand | February 2008-May 2011 | Sheep abattoir workers | 567 participants, 384 after follow-up (cases NR) | Incidence | None | New infection in hunters of goats, pigs or deer v no hunting of goats, pigs or deer OR 0.7 (95% CI: 0.1-2.5, p=0.636)  New infection in workers slaughtering of sheep, goats, pigs or deer at home v not slaughtering of sheep, goats, pigs or deer at home OR 0.8 (95% CI: 0.3-2.0, p=0.639) |
| [46] | New Zealand | May-October 2013 | Beef, sheep and deer farmers | 178 participants  12 seropositive | Seroprevalence | None | Seroprevalence in participants who perform any home slaughter v no home slaughter coefficient 0.94 (95% PI: 0.94-4.91, p=0.20)  Seroprevalence in participants who perform home slaughter beef v no home slaughter beef coefficient: -0.73 (95% PI: 2.29-0.55, p=0.13)  Seroprevalence in participants who perform home slaughter sheep v no home slaughter sheep coefficient: 1.52 (95% PI: 0.31-4.28, p=0.08)  Association between seroprevalence and hunting, home slaughter cattle, camping lakes/rivers and tramping reported NS (p-value NR) |
| [47] | New Zealand | May/June 2012 | Veterinarians | 277 participants  14 seropositive | Seroprevalence | None | **Seroprevalence in participants who perform home slaughter of cattle v no home slaughter of cattle OR 4.8 (95% CI: 1.5-15.2, p=0.02)** |
| [47] | New Zealand | May/June 2012 | Veterinarians | 277 participants  14 seropositive | Seroprevalence | Dog-cat exposure, home slaughter of pigs | **Seroprevalence in participants who perform home slaughter of cattle v no home slaughter of cattle OR 4.6 (95% CI: 1.3-16.1, Likelihood Ratio Test p=0.03)** |
| [47] | New Zealand | May/June 2012 | Veterinarians | 277 participants  14 seropositive | Seroprevalence | None | **Seroprevalence in participants who perform home slaughter of pigs v no home slaughter of pigs OR 8.5 (95% CI: 2.0-36.6, p=0.01)** |
| [47] | New Zealand | May/June 2012 | Veterinarians | 277 participants  14 seropositive | Seroprevalence | Dog-cat exposure, home slaughter of cattle | **Seroprevalence in participants who perform home slaughter of pigs v no home slaughter of pigs OR 7.9 (95% CI: 1.7-37.5, Likelihood Ratio Test p=0.02)** |
| [47] | New Zealand | May/June 2012 | Veterinarians | 277 participants  14 seropositive | Seroprevalence | None | **Seroprevalence in participants who perform home slaughter of sheep v no home slaughter of sheep OR 3.5 (95% CI: 1.2-10.2, Likelihood Ratio Test p=0.03)**  Seroprevalence in participants who perform home slaughter of deer v no home slaughter of deer reported NS (p-value NR)  Seroprevalence in participants hunting wild deer v not hunting wild deer OR 2.4 (95% CI 0.8-7.3, Likelihood Ratio Test p=0.16)  Seroprevalence in participants camping lakes/rivers v not camping lakes/rivers OR 2.5 (95% CI 0.8-7.6, Likelihood Ratio Test p=0.11) |
| [48] | Hawaii | July 1988-June 1989 (Big Island); July-December 1988 (Kauai) | Hospital patients | Big Island: 172 participants, 123 followed, 20 seropositive  Kauai: 100 participants, 59 followed, 13 cases  Inferential statistics: 33 cases, 77 controls | Incidence | None | **Frequency of handling animal tissues in cases v controls (p=0.005)** |
| **Presence of skin wounds** |  |  |  |  |  |  |  |
| [26] | Federated States of Micronesia | June-September 2011 | Hospital patients | 54 tested  11 confirmed cases | Incidence (confirmed cases only) | None | Case frequency in participants who reported wounds on legs/feet v no wounds on legs/feet OR 4.4 (95% CI: 1.1-17.8, p=0.056) |
| [48] | Hawaii | July 1988-June 1989 (Big Island); July-December 1988 (Kauai) | Hospital patients | Big Island: 172 participants, 123 followed, 20 seropositive  Kauai: 100 participants, 59 followed, 13 cases  Inferential statistics: 33 cases, 77 controls | Incidence | None | **Frequency of skin wounds in cases v controls (p=0.008)**  For those with skin wounds, cuts more strongly associated than abrasions (p=0.05) |
| **Smoking** |  |  |  |  |  |  |  |
| [22] | New Zealand | 1979-1980 | Meat inspectors and workers | Inspectors: 1215 sampled, 121 seropositive  Workers: 1248 sampled, 77 seropositive | Seroprevalence | None | Association between smoking and seroprevalence reported NS (p-value NR) |
| [46] | New Zealand | May-October 2013 | Beef, sheep and deer farmers | 178 participants  12 seropositive | Seroprevalence | None | Seroprevalence in participants smoking at work v not smoking at work coefficient 1.48 (95% PI:  -0.75-3.10, p=0.07) |
| [49] | New Zealand | Not reported | Pig farmers | 70 participants  20 seropositive | Seroprevalence | None | Association between smoking and seroprevalence reported NS (p-value NR) |

Note: statistically significant findings (defined by p<0.05 or if p-value not reported, then significance as reported by authors) presented in bold

Abbreviations: CI, confidence interval; NS, not significant; NR, not reported; OR, odds ratio; PI, probability interval; RR, relative risk

**Table S5:** Findings related to environmental drivers of human *Leptospira* infection

| **Citation details** | **Study location** | **Reported Study period** | **Study Population** | **Sample Size** | **Leptospirosis outcome** | **Covariates in model** | **Findings** |
| --- | --- | --- | --- | --- | --- | --- | --- |
| **Presence of animals in local environment** |  |  |  |  |  |  |  |
| [20] | Palau, Guam, Federated States of Micronesia, Vanuatu, Fiji, Tonga, Wallis, Futuna and French Polynesia | September 2003-December 2005 | Hospital patients | 263 suspected  69 confirmed cases | Incidence (confirmed cases only) | None | Case frequency in participants with contact with animals v no contact with animals reported NS at regional or island level (p-value NR) |
| [22] | New Zealand | 1979-1980 | Meat inspectors and workers | Inspectors: 1215 sampled, 121 seropositive  Workers: 1248 sampled, 77 seropositive | Seroprevalence | None | **Seroprevalence in workers with contact with pigs outside works v no contact with pigs outside works (p<0.01)** |
| [14] (Study 4) | New Caledonia (Coulée) | January 1985-December 1986 | General population | 93 sampled  16 seropositive | Seroprevalence | None | **Seropositive participants more frequented by murids v seronegative χ^2^(1)=9.32 (p<0.01)** |
| [26] | Federated States of Micronesia | June-September 2011 | Hospital patients | 54 tested  11 confirmed cases | Incidence (confirmed cases only) | None | Case frequency in participants with pigs around the home v no pigs around the home OR 0.3 (95% CI: 0.1–1.3, p= 0.187)  Case frequency in participants with dogs around the home v no dogs around the home OR 0.8 (95% CI: 0.2-3.2, p=0.730)  Case frequency in participants with rats around the home v no rats around the home rats OR 0.8 (95% CI: 0.2-3.8, p=1.000) |
| [27] | French Polynesia | March 2004-March 2005 | Hospital patients | 113 participants  33 cases (22 confirmed, 11 probable) | Incidence (confirmed or probable) | None | Contact with rats, dogs, pigs or horses in confirmed/suspected cases v unconfirmed cases reported p-value NS (p-value NR) |
| [32] | New Caledonia | January-June 2008 | Hospital patients | 135 cases (101 confirmed, 34 probable)  For inferential statistics: 98 cases, 410 controls | Incidence (confirmed or probable) | None | **Case frequency in participants with exposure to animals v no exposure to animals OR 2.04 (95% CI: 1.18-3.52) (p-value NR)**  **Case frequency in participants with exposure to cattle v no exposure to cattle OR 3.89 (95% CI: 2.18-6.92) (p-value NR)**  **Case frequency in participants with exposure to pigs v no exposure to pigs OR 2.12 (95% CI: 1.23-3.65) (p-value NR)**  **Case frequency in participants with exposure to horses v no exposure to horses OR 3.04 (95% CI: 1.75-5.26) (p-value NR)**  Case frequency in participants with exposure to dogs v no exposure to dogs OR 1.21 (95% CI: 0.76-1.93) (p-value NR)  **Case frequency in participants with rodents in/around house v no rodents in/around house OR 3.41 (95% CI: 2.05-5.66) (p-value NR)** |
| [38] | Fiji | September-December 2013 | General population | 2152 participants  417 seropositive | Seroprevalence | None | **Seroprevalence in participants with pigs at home/ garden v no pigs at home/garden OR 1.55 (95% CI: 1.23-2.12, p=0.007)**  Seroprevalence in participants with rats or mice around home v no rats or mice around home OR 1.16 (95% CI: 0.84-1.59, p=0.371)  **Seroprevalence in participants with physical contact with rats/mice v no physical contact with rats/mice OR 1.58 (95% CI: 1.20-2.09, p=0.001)**  Seroprevalence in participants with mongooses at/around home v no mongooses at/around home OR 1.08 (95% CI: 0.83-1.39, p=0.574)  **Seroprevalence in participants with physical contact with mongooses v no physical contact with mongooses OR 1.81 (95% CI: 1.23-2.68, p=0.003)**  **Seroprevalence in participants with cows at home/garden v no cows at home/garden OR 1.53 (95% CI: 1.15-2.05, p=0.004)**  **Seroprevalence in participants with cows in community v no cows in community OR 1.52 (95% CI: 1.19-1.93, p=0.001)**  **Seroprevalence in participants with horses at home/garden v no horses at home/garden OR 1.53 (95% CI: 1.09-2.14, p=0.013)**  **Seroprevalence in participants with horses in community v no horses in community OR 1.55 (95% CI: 1.19-2.01, p=0.001)**  Seroprevalence in participants with goats at home/garden v no goats at home/garden OR 1.08 (95% CI: 0.67-1.75, p=0.749)  **Seroprevalence in participants with goats in community v no goats in community OR 1.47 (95% CI: 1.08-2.01, p=0.015)**  Seroprevalence in participants with chickens at home/garden v no chickens at home/garden OR 1.21 (95% CI: 0.93-1.57, p=0.152)  **Seroprevalence in participants with chickens in community v no chickens in community OR 1.39 (95% CI: 1.12-1.72, p=0.003)**  Seroprevalence in participants with dogs at home/garden v no dogs at home/garden OR 1.00 (95% CI: 0.79-1.26, p=0.992)  **Seroprevalence in participants with dogs in community v no dogs in community OR 1.25 (95% CI 1.01-1.55, p=0.041)**  Seroprevalence in participants with cats at home/garden v no cats at home/garden OR 0.78 (95% CI: 0.58-1.06, p=0.115)  **Seroprevalence in participants with cats in community v no cats in community OR 1.38 (95% CI: 1.11-1.72, p=0.003)** |
| [38] | Fiji | September-December 2013 | General population | 2152 participants  417 seropositive | Seroprevalence | None | **Seroprevalence in participants with pigs in community v no pigs in community OR 1.81 (95% CI: 1.44-2.28) (p-value NR)** |
| [38] | Fiji | September-December 2013 | General population | 2152 participants  417 seropositive | Seroprevalence | Urban versus rural status, poverty rate, distance between home and river/creek, total cattle density, maximum rainfall in wettest month | **Seroprevalence in participants with pigs in community v no pigs in community OR 1.54 (95% CI: 1.21-1.98, p=0.001)** |
| [38] | Fiji | September-December 2013 | General population | 2152 participants  417 seropositive | Seroprevalence | None | Total cattle density in Tikina in seropositive participants v seronegative participants OR 1.02 (95% CI: 1.00-1.04) (p-value NR) |
| [38] | Fiji | September-December 2013 | General population | 2152 participants  417 seropositive | Seroprevalence | Urban versus rural status, poverty rate, distance between home and river/creek, pigs in community, maximum rainfall in wettest month | **Total cattle density in Tikina in seropositive participants v seronegative participants OR 1.04 (95% CI: 1.02-1.06, p<0.001)** |
| [43] | Fiji | 2013 | General population | 2046 participants (number seropositive NR) | Seroprevalence | Maximum rainfall in wettest month, distance to river, poverty rate, rural residential setting | **Seroprevalence based on cattle density per squared kilometre significant for Logistic Regression model (p-value NR)** |
| [44] | Vanuatu | January 2013-August 2014 | Hospital patients | 161 patients  12 cases (5 confirmed, 7 probable)  29 seropositive | Incidence (confirmed or probable cases), Seroprevalence | None | Seroprevalence in participants in contact with dogs/cats v no contact with dogs/cats OR 0.45 (95% CI: 0.11-1.87, p=0.27)  Seroprevalence in participants in contact with horses, goats or sheep v no contact with horses, goats or sheep OR 2.86 (95% CI: 0.66-12.32, p=0.159)  Seroprevalence in participants with rodents seen in vicinity v no rodents seen in vicinity OR 0.85 (95% CI: 0.17-4.25, p=0.85) |
| [44] | Vanuatu | January 2013-August 2014 | Hospital patients | 161 patients  12 cases (5 confirmed, 7 probable)  29 seropositive | Incidence (confirmed or probable cases), Seroprevalence | None | **Seroprevalence in participants in contact with pigs v no contact with pigs OR 3.52 (95% CI: 1.49-8.30, p=0.004)** |
| [44] | Vanuatu | January 2013-August 2014 | Hospital patients | 161 patients  12 cases (5 confirmed, 7 probable)  29 seropositive | Incidence (confirmed or probable cases), Seroprevalence | Sex, water at home, freshwater fishing, contact with surface water, hunting, contact with animals (dogs/ cats, horses/ goats/ sheep, cattle), rodents seen in vicinity, island of residence, occupational risk | Seroprevalence in participants in contact with pigs v no contact with pigs OR 2.68 (95% CI: 0.87-8.27, p=0.09) |
| [44] | Vanuatu | January 2013-August 2014 | Hospital patients | 161 patients  12 cases (5 confirmed, 7 probable)  29 seropositive | Incidence (confirmed or probable cases), Seroprevalence | None | **Seroprevalence in participants in contact with cattle v no contact with cattle OR 5.60 (95% CI: 1.68-18.65, p=0.005)** |
| [44] | Vanuatu | January 2013-August 2014 | Hospital patients | 161 patients  12 cases (5 confirmed, 7 probable)  29 seropositive | Incidence (confirmed or probable cases), Seroprevalence | Sex, water at home, freshwater fishing, contact with surface water, hunting, contact with animals (pigs, dogs/ cats, horses /goats/ sheep), rodents seen in vicinity, island of residence, occupational risk | Seroprevalence in participants in contact with cattle v no contact with cattle OR 2.37 (95% CI: 0.48-11.70, p=0.30) |
| [45] | New Caledonia | 1989 | General population | Total population 165000  144 cases | Incidence | None | No association reported between serogroups and animal contact (p-value NR) |
| [46] | New Zealand | May-October 2013 | Beef, sheep and deer farmers | 178 participants  12 seropositive | Seroprevalence | None | Seroprevalence in participants with pet dog in house v no pet dog in house coefficient 1.29 (95% PI: -0.16-3.36, p=0.05)  Seroprevalence in participants with cat in house v no cat in house coefficient 0.95 (95% PI: -0.50-2.97, p=0.13)  Seroprevalence in participants with dairy cattle exposure v no dairy cattle exposure coefficient: 0.65 (95% PI: -0.65 to 1.86, p=0.14)  **Seroprevalence in participants with goat exposure v no goat exposure coefficient= -2.33 (95% PI -3.96 to -0.59, p=0.01)**  **Seroprevalence in participants with high rabbit abundance on farm v low rabbit abundance on farm coefficient -1.22 (95% PI: -2.81-0.12, p=0.04)**  Seroprevalence in participants with livestock exposure outside farm v no livestock exposure outside farm reported NS (p-value NR)  Association between hedgehog, mice, wild pig, rat or wild cat abundance on farm and seroprevalence in participants reported NS (p-value NR) |
| [46] | New Zealand | May-October 2013 | Beef, sheep and deer farmers | 178 participants  12 seropositive | Seroprevalence | None | **Seroprevalence in participants with high wild deer abundance on farm v low wild deer abundance on farm OR 1.53 (95% PI: 0.21-2.83, p=0.02)** |
| [46] | New Zealand | May-October 2013 | Beef, sheep and deer farmers | 178 participants  12 seropositive | Seroprevalence | Assisting calving/fawning, species farmed flat terrain on farm, possum abundance on farm | **Seroprevalence in participants with high wild deer abundance on farm v low wild deer abundance on farm coefficient 2.52 (median prevalence ratio 10.8, 95% PI: 2.4-57.0) (p-value NR)** |
| [46] | New Zealand | May-October 2013 | Beef, sheep and deer farmers | 178 participants  12 seropositive | Seroprevalence | None | **Seroprevalence in participants with high possum abundance on farm v low possum abundance on farm OR -1.94 (95% PI: -6.04—0.03, p=0.02)** |
| [46] | New Zealand | May-October 2013 | Beef, sheep and deer farmers | 178 participants  12 seropositive | Seroprevalence | Assisting calving/fawning, wild deer abundance on farm, species farmed flat terrain on farm | **Seroprevalence in participants with high possum abundance on farm v low possum abundance on farm coefficient= -2.42 (median prevalence ratio 0.1, 95% PI: 0.0-0.7) (p-value NR)** |
| [47] | New Zealand | May/June 2012 | Veterinarians | 277 participants  14 seropositive | Seroprevalence | None | Seroprevalence in participants who own deer v do not own deer OR 3.2 (95% CI: 0.7-15.8, p=0.20)  Seroprevalence in participants who own pigs v do not own pigs OR 3.0 (95% CI: 0.6-13.6, p=0.23)  Seroprevalence in participants owning cattle, sheep, horses, dogs or cats v do not own cattle, sheep, horses, dogs or cats (Likelihood Ratio Test p>0.35) |
| [51] | New Zealand | 1990-1998 | Notification data | Total population NR  1397 cases | Incidence | None | **Positive linear correlation between leptospirosis incidence and ratio of dairy cattle numbers to human population in each territorial authority area (R^2=0.28, F=28.0, p<0.0001)** |
| **Seasonality** |  |  |  |  |  |  |  |
| [18] | Hawaii | 1970-1984 | Notification data | Total population NR  195 cases | Incidence | None | Difference in incidence when grouped by month of onset reported NS (p>0.05) |
| [28] | New Zealand | January 2004-December 2010 | Notification data | 97 cases (86 confirmed, 8 probable, 3 pending) | Incidence (confirmed or probable or pending) | None | **Significant difference between rate of cases during the winter v non-winter months (p<0.05)** |
| [34] | New Caledonia | January 2006-December 2016 | Notification data | Total population 268767 904 cases (700 confirmed, 204 probable) | Incidence (confirmed or probable) | None | **Patients <15 years old had a lower incidence rate from March to December v other months RR 0.31 (95% CI: 0.24-0.40, p<0.0001)** |
| [48] | Hawaii | July 1988-June 1989 (Big Island); July-December 1988 (Kauai) | Hospital patients | Big Island: 172 participants, 123 followed, 20 seropositive  Kauai: 100 participants, 59 followed, 13 cases  Inferential statistics: 33 cases, 77 controls | Incidence | None | No difference between cases and controls based on season of onset (p<0.47) |
| **Rainfall/ floods** |  |  |  |  |  |  |  |
| [38] | Fiji | September-December 2013 | General population | 2152 participants  417 seropositive | Seroprevalence | None | **Maximum rainfall in wettest month (per mm) in seropositive participants v seronegative participants OR 1.003 (95% CI: 1.001-1.005) (p-value NR)** |
| [38] | Fiji | September-December 2013 | General population | 2152 participants  417 seropositive | Seroprevalence | Urban/rural, poverty rate, distance between home and river or major creek, presence of pigs in community, total cattle density in Tikina | **Maximum rainfall in wettest month (per mm) in seropositive participants v seronegative participants OR 1.04 (95% CI: 1.02-1.06, p<0.001)** |
| [42] | Futuna | 2004-2014 | Notification data | Total population 3612  382 cases (confirmed/ probable not specified) | Incidence (confirmed or probable) | None | **Cases (probable and confirmed) were correlated with rainfall observed 2 months earlier (R^2^=0.569) (p-value NR)**  Association between total annual rainfall and incidence (using only confirmed cases) reported NS (R^2^=0.13) (p-value NR) |
| [43] | Fiji | 2013 | General population | 2046 participants (number seropositive NR) | Seroprevalence | Cattle density, distance to river, poverty rate, rural residential setting | **Association between seroprevalence and maximum rainfall in wettest month (mm) using Geographically Weighted Logistic Regression model median OR 1.30 (IQR 1.27–1.35) (p-value NR)**  **Association between seroprevalence and maximum rainfall in wettest month (mm) using Logistic Regression model OR 1.26 (95% CI 1.09–1.45) (p-value NR)** |
| [48] | Hawaii | July 1988-June 1989 (Big Island); July-December 1988 (Kauai) | Hospital patients | Big Island: 172 participants, 123 followed, 20 seropositive  Kauai: 100 participants, 59 followed, 13 cases  Inferential statistics: 33 cases, 77 controls | Incidence | None | Mean total rainfall for cases (16.3 inches) v controls (12.4 inches) (p<0.16) |
| [52] | Fiji | December 2011-May 2012 | People who sought medical care | Total population 340000  1217 suspected cases (31 confirmed, 283 probable) | Incidence (confirmed or probable) | None | **Higher mean weekly case count during flood-associated (10 weeks) v not flood-associated (11 weeks) periods risk ratio 3.37 (95% CI: 3.24- 3.51) (p-value NR)**  **Quantitative polymerase chain reaction positivity rate higher among flood-associated cases v non-flood-associated cases (p=0.02)** |
| **Location** |  |  |  |  |  |  |  |
| [21] | New Zealand | July-November 1978 | Meat inspectors | 1003 sampled  103 seropositive | Seroprevalence | None | **Significant difference between seroprevalence rate of inspectors working in North Island v South Island (p<0.01)** |
| [24] | French Polynesia | January 2007-December 2017 | General population | Total population 280000 1365 cases (851 confirmed, 505 probable) | Incidence (annual incidence rates using confirmed and probable cases) | None | **Annual incidence rates in Moorea-Maiao v Tahiti RR 1.6 (95% CI: 1.3-1.9, p<0.001)**  **Annual incidence rates in Raiatea v Tahiti RR 3.6 (95% CI: 3.0-4.2, p<0.001)**  **Annual incidence rates in Tahaa v Tahiti RR 4.1 (95% CI: 3.3-5.1, p<0.001)**  **Annual incidence rates in Hauhine v Tahiti RR 3.1 (95%: CI: 2.5-3.9, p<0.001)**  Annual incidence rates in Bora-Bora v Tahiti RR 0.9 (95% CI: 0.6-1.2, p=0.437)  Annual incidence rates in Maupiti v Tahiti RR 0.2 (95% CI: 0.03-1.4, p=0.098) |
| [25] | New Caledonia | January 1985-December 1986 | General population | Total population 145368  193 cases | Incidence | None | **Cases in Nera v rest of the country χ^2^(1)=496.08 (p<0.05)** |
| [14] (Study 1, Study 3) | New Caledonia (Nera, Coulée) | January 1985-December 1986 | General population | Nera: 3140 total population, 60 cases  Coulée: 14614 total population, 26 cases | Incidence | None | **Incidence in Nera basin v rest of the entire territory excluding Coulée χ^2^(1)=116.26 (p<0.001)**  **Incidence in Coulée v rest of the entire territory excluding Nera basin χ^2^(1)=6.65 (p<0.001)**  **Cases in Nera basin v Coulée χ^2^(1) =143.39 (p<0.001)** |
| [27] | French Polynesia | March 2004-March 2005 | Hospital patients | 113 participants  33 cases (22 confirmed, 11 probable) | Incidence (confirmed or probable) | None | Higher incidence in Raiatea v the Marquesas but reported NS (p-value NR) |
| [31] | New Zealand | 2010-2015 | General population | Total population NR  442 cases | Incidence | None | Average incidence rate in North Island v South Island (p=0.57) |
| [33] | Hawaii | January 1974-December 1998 | General population | Total population NR 709 cases (353 confirmed, 180 probable, 176 suspected) | Incidence (confirmed only) | None | **Mean annual incidence rate in Hawaii v Oahu RR 18.26 (95% CI: 13.83-24.32, p<0.0001)**  **Mean annual incidence rate in Kauai v Oahu RR 24.50 (95% CI: 18.00-33.50, p<0.0001)** |
| [33] | Hawaii | January 1974-December 1998 | General population | Total population NR 709 cases (353 confirmed, 180 probable, 176 suspected) | Incidence (confirmed only) | Age group, ethnicity | **Confirmed cases in Hawaii v Oahu OR 17.30 (95% CI: 11.23-27.50, p<0.0001)**  **Confirmed cases in Kauai v Oahu OR 27.61 (95% CI: 17.27-45.09, p<0.0001)** |
| [37] | American Samoa | May-July 2010 | General population | 807 participants  125 seropositive | Seroprevalence | None | **Serovar LT 751 (serogroup Australis): significantly more common on Manu'a Islands (Ta'u Ofu and Olosega); seroprevalence in Manu'a residents v Tutuila residents OR 2.53 (95% CI: 1.07-5.98, p=0.03)**  Serovar LT1163 (serogroup Pyrogenes): only found on Tutuila and Ta’u but reported NS between islands (p-value NR)  **Serovar Hebdomadis (serogroup Hebdomadis): only found on Tutuila and significant difference in seroprevalence in Tutuila v other islands (p=0.001)** |
| [38] | Fiji | September-December 2013 | General population | 2152 participants  417 seropositive | Seroprevalence | None | **Seroprevalence in settlements v urban residential community types OR 2.25 (95% CI: 1.55-3.26) (p-value NR)**  **Seroprevalence in villages v urban residential community types OR 3.56 (95% CI: 2.54-5.00) (p-value NR)** |
| [38] | Fiji | September-December 2013 | General population | 2152 participants  417 seropositive | Seroprevalence | Gender, ethnicity, metered water available at home and work location | **Seroprevalence in settlements v urban residential community types OR 2.13 (95% CI: 1.41-3.21, p<0.001)**  **Seroprevalence in villages v urban residential community types OR 1.64 (95% CI 1.08-2.51, p=0.021)** |
| [38] | Fiji | September-December 2013 | General population | 2152 participants  417 seropositive | Seroprevalence | None | **Seroprevalence in rural v urban/peri-urban communities OR 2.22 (95% CI: 1.75-2.82) (p-value NR)** |
| [38] | Fiji | September-December 2013 | General population | 2152 participants  417 seropositive | Seroprevalence | Poverty rate, distance between home and river/creek, presence of pigs in community, total cattle density, maximum rainfall in wettest month | **Seroprevalence in rural v urban/peri-urban communities OR 1.43 (95% CI 1.07-1.91, p=0.016)** |
| [41] | Wallis and Futuna | January 2008-June 2015 | Hospital patients | 338 suspected, 165 confirmed and 173 excluded cases | Incidence (suspected or confirmed) | None | **Island of origin not Futuna seroconversion v no seroconversion RR 0.23 (p<0.02)** |
| [42] | Futuna | 2004-2014 | Notification data | Total population 3612  382 cases (confirmed/ probable not specified) | Incidence (confirmed or probable) | None | **2004-2008: significant difference in cases in Poi v other locations RR 2.77 (Log Likelihood Ratio 6.32, p=0.01); significant difference in cases in Taoa v other locations RR 1.95 (Log Likelihood Ratio 5.05, p=0.03)**  2008-2009: no clusters of cases based on location (p-value NR)  **2010-2014: significant difference in cases in Laeva-Nuku-Vaisei v other locations RR 2.07 (Log Likelihood Ratio 6.64, p=0.004)**  **2004-2014: significant difference in cases in Laeva-Nuku-Vaisei-Taoa v other locations RR 1.48 (Log Likelihood Ratio 7.08, p=0.002)** |
| [43] | Fiji | 2013 | General population | 2046 participants (number seropositive NR) | Seroprevalence | Cattle density, maximum rainfall in wettest month, distance to river, poverty rate | **Significantly higher seroprevalence in rural residential setting v urban/peri-urban using Logistic Regression model (p-value NR)** |
| [44] | Vanuatu | January 2013-August 2014 | Hospital patients | 161 patients  12 cases (5 confirmed, 7 probable)  29 seropositive | Incidence (confirmed or probable cases), Seroprevalence | None | **Seroprevalence in participants living in island other than Efate v living in Efate OR 2.34 (95% CI: 1.02-5.37, p=0.04)** |
| [44] | Vanuatu | January 2013-August 2014 | Hospital patients | 161 patients  12 cases (5 confirmed, 7 probable)  29 seropositive | Incidence (confirmed or probable cases), Seroprevalence | Sex, water at home, freshwater fishing, contact with surface water, hunting, contact with animals (pigs, dogs/ cats, horses /goats/ sheep, cattle), rodents seen in vicinity, occupational risk | Seroprevalence in participants living in island other than Efate v living in Efate OR 1.59 (95% CI: 0.51-4.99, p=0.43) |
| [46] | New Zealand | May-October 2013 | Beef, sheep and deer farmers | 178 participants  12 seropositive | Seroprevalence | None | Association between island and seroprevalence in participants reported NS (p-value NR) |
| [48] | Hawaii | July 1988-June 1989 (Big Island); July-December 1988 (Kauai) | Hospital patients | Big Island: 172 participants, 123 followed, 20 seropositive  Kauai: 100 participants, 59 followed, 13 cases  Inferential statistics: 33 cases, 77 controls | Incidence | None | No difference reported between cases and controls based on island (p<0.93) |
| [50] | Palau | May 2000- June 2006 | General population | Total population 20000  81 cases | Incidence | None | Incidence in urban v rural states (F-value 1.39, F p-value=0.38, pooled T-value -0.55, p=0.59) |
| **Geographical characteristics of household** |  |  |  |  |  |  |  |
| [35] | American Samoa | May-July 2010 | General population | 807 participants (number seropositive NR) | Seroprevalence | None | Seroprevalence in participants with house below median altitude of village v house not below median altitude of village OR 1.47 (95% CI: 0.96-2.27) (p-value NR) |
| [35] | American Samoa | May-July 2010 | General population | 807 participants (number seropositive NR) | Seroprevalence | Male, occupational group, vegetation type, soil type, piggeries within 250 metres and above house | **Seroprevalence in participants with house below median altitude of village v house not below median altitude of village OR 1.58 (95% CI: 1.00-2.49) (p-value NR)** |
| [35] | American Samoa | May-July 2010 | General population | 807 participants (number seropositive NR) | Seroprevalence | None | **Seroprevalence in participants with piggeries within 250 metres and above house OR 1.16 (95% CI: 1.07-1.26) (p-value NR)** |
| [35] | American Samoa | May-July 2010 | General population | 807 participants (number seropositive NR) | Seroprevalence | Male, occupational group, house below median altitude of village, vegetation type, soil type | **Seroprevalence in participants with piggeries within 250 metres and above house OR 1.15 (95% CI: 1.05-1.26) (p-value NR)** |
| [36] | American Samoa | May-July 2010 | General population | 807 participants  125 seropositive | Seroprevalence | None | **Seroprevalence in participants with house below median altitude of village v house not below median altitude of village OR 1.53 (95% CI: 1.03-2.28) (p-value NR)** |
| [36] | American Samoa | May-July 2010 | General population | 807 participants  125 seropositive | Seroprevalence | None | **Seroprevalence in participants with >5 piggeries within 250 metres and above house v 0-2 piggeries OR 2.63 (95% CI: 1.52-4.440) (p-value NR)**  Seroprevalence in participants with 3-5 piggeries within 250 metres and above house v 0-2 piggeries OR 1.34 (95% CI: 0.85-2.12) (p-value NR) |
| [36] | American Samoa | May-July 2010 | General population | 807 participants  125 seropositive | Seroprevalence | Male, heard of leptospirosis, occupation | **Seroprevalence in participants with >5 piggeries within 250 metres and above house v 0-2 piggeries OR 2.66 (95% CI: 1.55-4.57) (p-value NR)**  Seroprevalence in participants with 3-5 piggeries within 250 metres and above house v 0-2 piggeries 1.44 (95% CI: 0.90-2.33) (p-value NR) |
| [38] | Fiji | September-December 2013 | General population | 2152 participants  417 seropositive | Seroprevalence | None | **Seroprevalence in participants with distance between home and river/major creek <= 100 metres v distance >100 metres OR 1.58 (95% CI: 1.24-2.03) (p-value NR)** |
| [38] | Fiji | September-December 2013 | General population | 2152 participants  417 seropositive | Seroprevalence | Urban/rural, poverty rate, presence of pigs in community, total cattle density in Tikina, maximum rainfall in wettest month | **Seroprevalence in participants with distance between home and river/major creek <= 100 metres v distance >100 metres OR 1.41 (95% CI 1.09-1.83, p=0.009)** |
| [43] | Fiji | 2013 | General population | 2046 participants (number seropositive NR) | Seroprevalence | Cattle density, maximum rainfall in wettest month, poverty rate, rural residential setting | **Association between seroprevalence and distance to river <100 metres using Geographically Weighted Logistic Regression OR 1.45 (IQR 1.35–2.05) (p-value NR)**  **Association between seroprevalence and distance to river <100 metres using Logistic Regression OR 1.61 (95% CI: 1.24–2.18) (p-value NR)** |
| **Soil/ vegetation type** |  |  |  |  |  |  |  |
| [35] | American Samoa | May-July 2010 | General population | 807 participants (number seropositive NR) | Seroprevalence | None | Seroprevalence in participants with urban cultivated vegetation type v urban built up OR 1.22 (95% CI: 0.74-1.99) (p-value NR)  **Seroprevalence in participants with** **agricultural vegetation type v urban built up OR 2.33 (95% CI: 1.28-4.23) (p-value NR)**  Seroprevalence in participants with other vegetation type v urban built up OR 2.21 (95% CI: 0.69-7.07) (p-value NR) |
| [35] | American Samoa | May-July 2010 | General population | 807 participants (number seropositive NR) | Seroprevalence | May include male, occupational group, house below median altitude of village, soil type and piggeries within 250 metres and above house | Seroprevalence in participants with urban cultivated vegetation type v urban built up OR 1.13 (95% CI: 0.67-1.88) (p-value NR)  **Seroprevalence in participants with** **agricultural vegetation type v urban built up OR 2.09 (95% CI: 1.12-3.89)** **(p-value NR)**  Seroprevalence in participants with other vegetation type v urban built up OR 1.66 (95% CI: 0.49-5.61) (p-value NR) |
| [35] | American Samoa | May-July 2010 | General population | 807 participants (number seropositive NR) | Seroprevalence | None | **Seroprevalence in participants with clay loam soil type v clay OR 3.11 (95% CI: 1.27-7.61) (p-value NR)**  Seroprevalence in participants with urban soil type v clay OR 2.04 (95% CI: 0.81-5.10) (p-value NR)  Seroprevalence in participants with other soil type v clay OR 2.20 (95% CI: 0.79-6.14) (p-value NR) |
| [35] | American Samoa | May-July 2010 | General population | 807 participants (number seropositive NR) | Seroprevalence | May include male, occupational group, house below median altitude of village, vegetation type, piggeries within 250 metres and above house | **Seroprevalence in participants with clay loam soil type v clay OR 2.72 (95% CI: 1.08-6.85**) **(p-value NR)**  Seroprevalence in participants with urban soil type v clay OR 1.86 (95% CI 0.72-4.78) (p-value NR)  Seroprevalence in participants with other soil type v clay OR 2.09 (95% CI 0.73-5.98) (p-value NR) |
| [37] | American Samoa | May-July 2010 | General population | 807 participants  125 seropositive | Seroprevalence | None | **Tutuila:**  **Residents in agricultural areas more likely seropositive v urban-built up areas – serovar Hebdomadis (serogroup Hebdomadis) OR 3.04 (p=0.001), serovar LT 751 (serogroup Australis) OR 3.31 (p=0.01)**  Serovar LT 1163 (serogroup Pyrogenes) more common in 'other' vegetation types v urban built up but reported NS (p-value NR) |
| **Population density** |  |  |  |  |  |  |  |
| [37] | American Samoa | May-July 2010 | General population | 807 participants  125 seropositive | Seroprevalence | None | Association between population density and overall seroprevalence reported NS (p=0.51)  Association between population density and the prevalence of serovar Hebdomadis (serogroup Hebdomadis) reported NS (p=0.60)  **Distribution of serovar LT 1163 (serogroup Pyrogenes) differed significantly between population density areas (p=0.001)** |

Note: statistically significant findings (defined by p<0.05 or if p-value not reported, then significance as reported by authors) presented in bold

Abbreviations: CI, confidence interval; NS, not significant; NR, not reported; OR, odds ratio; PI, probability interval; RR, relative risk
